# Cataloging SCN resistance loci in North American public soybean breeding programs

**DOI:** 10.3389/fpls.2023.1270546

**Published:** 2023-11-20

**Authors:** Anser Mahmood, Kristin D. Bilyeu, Mária Škrabišová, Jana Biová, Elizabeth J. De Meyer, Clinton G. Meinhardt, Mariola Usovsky, Qijian Song, Aaron J. Lorenz, Melissa G. Mitchum, Grover Shannon, Andrew M. Scaboo

**Affiliations:** ^1^ Division of Plant Science and Technology, University of Missouri, Columbia, MO, United States; ^2^ Plant Genetics Research Unit, United States Department of Agriculture-Agricultural Research Service, University of Missouri, Columbia, MO, United States; ^3^ Department of Biochemistry, Faculty of Science, Palacky University Olomouc, Olomouc, Czechia; ^4^ Soybean Genomics and Improvement Laboratory, Beltsville Agricultural Research Center, United States Department of Agriculture-Agricultural Research Service (USDA-ARS), Beltsville, MD, United States; ^5^ Department of Agronomy and Plant Genetics, University of Minnesota, St. Paul, MN, United States; ^6^ Department of Plant Pathology and Institute of Plant Breeding, Genetics, and Genomics, University of Georgia, Athens, GA, United States

**Keywords:** soybean (*Glycine max* (L.) Merr.), SCN (*Heterodera glycines* Ichinohe), resistance to *Heterodera glycines* 1 (*Rhg1*), genome wide association study (GWAS), α-soluble *N*-ethylmaleimide sensitive factor attachment protein (α-SNAP)

## Abstract

Soybean cyst nematode (SCN) is a destructive pathogen of soybeans responsible for annual yield loss exceeding $1.5 billion in the United States. Here, we conducted a series of genome-wide association studies (GWASs) to understand the genetic landscape of SCN resistance in the University of Missouri soybean breeding programs (Missouri panel), as well as germplasm and cultivars within the United States Department of Agriculture (USDA) Uniform Soybean Tests—Northern Region (NUST). For the Missouri panel, we evaluated the resistance of breeding lines to SCN populations HG 2.5.7 (Race 1), HG 1.2.5.7 (Race 2), HG 0 (Race 3), HG 2.5.7 (Race 5), and HG 1.3.6.7 (Race 14) and identified seven quantitative trait nucleotides (QTNs) associated with SCN resistance on chromosomes 2, 8, 11, 14, 17, and 18. Additionally, we evaluated breeding lines in the NUST panel for resistance to SCN populations HG 2.5.7 (Race 1) and HG 0 (Race 3), and we found three SCN resistance-associated QTNs on chromosomes 7 and 18. Through these analyses, we were able to decipher the impact of seven major genetic loci, including three novel loci, on resistance to several SCN populations and identified candidate genes within each locus. Further, we identified favorable allelic combinations for resistance to individual SCN HG types and provided a list of available germplasm for integration of these unique alleles into soybean breeding programs. Overall, this study offers valuable insight into the landscape of SCN resistance loci in U.S. public soybean breeding programs and provides a framework to develop new and improved soybean cultivars with diverse plant genetic modes of SCN resistance.

## 1 Introduction

Soybean cyst nematode (SCN; *Heterodera glycines* Ichinohe) is the number one pathogen of soybeans responsible for annual yield loss exceeding $1.5 billion in the United States (Bandara et al., 2020). SCN populations are classified by HG type (HG = *H. glycines*) (formerly race designations) based on their reproductive potential on a set of soybean indicator lines compared to a susceptible check (Howland et al., 2018). The former race system initially classified SCN populations into 16 races based on their ability to reproduce on four differential lines, including Pickett, plant introduction (PI) 548402 (‘Peking’), PI 88788, and PI 90763 (Riggs and Schmitt, 1988). The race classification system was later expanded to include PI 437654 as a measure of SCN parasitism owing to concerns regarding the utility of this system in the characterization of resistance (Schmitt and Shannon, 1992). The race system was updated to the HG type test in 2002, where an SCN population is assigned a numerical designation based on the indicator line number to which it is virulent (female index >10) using a set of seven indicator lines, including 1) PI 548402, 2) PI 88788, 3) PI 90763, 4) PI 437654, 5) PI 209332, 6) PI 89772, and 7) PI 548316 (‘Cloud’) (Niblack et al., 2002).

Planting resistant soybean cultivars is a cost-effective and eco-friendly strategy for SCN management (Mitchum, 2016). Multiple studies have investigated the genetic mechanisms of SCN resistance over the last six decades, and 216 QTL regions have been identified and cataloged at SoyBase.org to date (www.soybase.com, 2023). Two major genes and their alleles, *Rhg1* and *Rhg4* (resistance to *H. glycines* 1 and 4), have been utilized by public and private soybean breeders for the development of SCN resistance cultivars. The *Rhg1* locus has been shown to harbor a tandem repeat of three genes in multiple copies, including an α-soluble *N*-ethylmaleimide-sensitive factor attachment protein gene (*GmSNAP18*; *Glyma.18g022500*) (Cook et al., 2012; Lee et al., 2015). Two resistant haplotypes of *Rhg1*, namely, *rhg1-b* and *rhg1-a*, have been previously identified and shown to carry slightly different versions of α-soluble *N*-ethylmaleimide-sensitive factor attachment protein (α-SNAP) encoded by *GmSNAP18* (Cook et al., 2014). The abnormal α-SNAP proteins encoded by these resistant haplotypes have been demonstrated to impart resistance through poisoning of the SCN syncytia (Bayless et al., 2016). The *Rhg4* locus, in contrast, has been shown to encode a cytosolic serine hydroxymethyl transferase gene (*GmSHMT08*; *Glyma.08g108900*) (Liu et al., 2012). The resistant *Rhg4* allele has been shown to harbor two single-nucleotide polymorphism (SNP) substitutions that alter the function of the encoded protein (Liu et al., 2012). SCN resistance gene sources have been classified into PI 88788- and Peking-type resistance based on these two loci and their allelic/haplotype variants (Bayless et al., 2019). The PI 88788-type resistance is attributed to the *rhg1-b* haplotype at the *Rhg1* locus. Contrastingly, Peking-type resistance governs an epistatic effect between the *rhg1-a* haplotype at the *Rhg1* locus and the *Rhg4* locus (Liu et al., 2012; Liu et al., 2017), along with an additional epistatic interaction between *rhg1-a* and the SCN resistance locus *rhg2* (Basnet et al., 2022).

It has been shown that the α-soluble *N*-ethylmaleimide-sensitive factor attachment protein gene (*GmSNAP11*; *Glyma.11g234500*) located at the *Rhg2* locus contributes a role in Peking-type SCN resistance (Lakhssassi et al., 2017; St-Amour et al., 2020; Suzuki et al., 2020; Basnet et al., 2022; Shaibu et al., 2022). The resistant *Rhg2* allele carries a splice site variant that induces a premature stop codon resulting in low protein abundance (Matsye et al., 2012; Bayless et al., 2018). In addition to *GmSNAP18* and *GmSNAP11*, soybean harbors three *GmSNAP*s on chromosomes 2, 9, and 14. *GmSNAP02* has now been shown to confer resistance to HG 1.2.5.7 populations through a loss-of-function (Usovsky et al., 2023). *GmSNAP14* has previously been reported to not play a role in SCN resistance (Lakhssassi et al., 2017), while the function of *GmSNAP09* has not been reported. Additional investigations into the molecular mechanisms responsible for resistance governed by *rhg1-a* and *rhg1-b* have pointed to an unusual allele of the *N*-ethylmaleimide-sensitive factor gene (*GmNSF_RAN07_*; *Glyma.07g195900*) on chromosome 7, which is essential for viability of soybeans carrying these resistance loci (Bayless et al., 2018).

Traditionally, bi-parental quantitative trait locus (QTL) mapping studies have been successfully used to elucidate the genetic regions associated with resistance to different SCN populations (Guo et al., 2005; Guo et al., 2006; Wu et al., 2009; Vuong et al., 2021). Despite the widespread utilization of bi-parental QTL mapping and its success in trait discovery, this method suffers from low genomic resolution due to limited recombination events and the ability to only capture the polymorphisms between the two parents (Zhu et al., 2008). Genome-wide association studies (GWASs) are an efficient complementary approach to QTL mapping, which allow the determination of marker–trait associations in genetically unstructured accession panels (Zhu et al., 2008). In soybeans, GWASs have been successfully employed to validate *Rhg1* and *Rhg4* SCN resistance loci (Han et al., 2015; Vuong et al., 2015; Tran et al., 2019; Tian et al., 2023). Further, quantitative trait nucleotides (QTNs) on chromosomes 18 and 19 have been identified in *Glycine soja* accessions for resistance to HG 2.5.7 (Race 5) using a GWAS (Zhang et al., 2016). A similar analysis using *G. soja* accessions identified QTNs on chromosomes 4, 9, and 16 for resistance to HG 2.5.7 (Race 1) (Zhang et al., 2017). Investigations into resistance for HG 0 (Race 3) using a GWAS have identified QTNs on chromosomes 7 and 10 (Tran et al., 2019). A recent study using a GWAS identified *Rhg1*, *Rhg2*, *GmNSF_RAN07_*, and a QTN on chromosome 7 for resistance to HG 0 (Tian et al., 2023). These studies demonstrate the effectiveness of GWASs in identifying marker–trait associations for complex traits such as SCN resistance.

Despite the discovery of multiple loci in previous studies, most cultivars grown in the United States have been reported to utilize the *rhg1-b* allele for SCN resistance (McCarville et al., 2016; McCarville et al., 2017; Rincker et al., 2017; Howland et al., 2018; Chowdhury et al., 2021). The limited application of other major and minor effect QTLs in public and private breeding programs could be attributed to issues associated with the introgression of these loci. This is especially true for minor effect loci where the use of these loci is hindered by the time-consuming nature of backcrossing and potential linkage drag associated with trait introgression. Furthermore, excessive use of *rhg1-b* over long periods has led to a shift in SCN populations, where a widespread increase in HG 2.- and 1.2.- populations have been observed (Howland et al., 2018; Chowdhury et al., 2021).

In this study, we aimed to catalog the resistance loci for individual SCN populations by conducting GWASs with experimental lines and cultivars from the University of Missouri soybean breeding programs (Missouri panel) and with breeding lines and cultivars from within the United States Department of Agriculture (USDA) Uniform Soybean Tests—Northern Region (NUST panel). Compared to the USDA Soybean Germplasm Collection, which consists of 20,087 genotyped wild and cultivated soybean accessions, these two panels consisted solely of breeding lines and cultivars from Missouri and other breeding programs from the northern and mid-western states of the USA and provinces of Canada. Due to decades of breeding for SCN resistance, these two panels have a higher frequency of SCN resistance alleles than the USDA Soybean Germplasm Collection. Hence, GWASs using these panels enabled us to understand the landscape of resistance loci in these two panels and to decipher the novel sources of SCN resistance with a higher resolution. The major and minor QTNs identified in this study offer great potential in selective and precise breeding for SCN resistance. Hence, this study provides solutions to diversify commercially available soybean cultivars with novel sources of SCN resistance.

## 2 Materials and methods

### 2.1 Plant material and SCN bioassay

Two different GWAS panels were used for analysis in this study. The first panel consisted of advanced breeding lines and cultivars from the University of Missouri’s northern and southern soybean breeding programs (Missouri panel). The lines and cultivars in this panel were generated by the University of Missouri’s soybean breeding programs as part of routine breeding efforts for the development of high-quality soybean cultivars. To screen for SCN resistance, these lines were phenotyped with five SCN inbred populations: HG 2.5.7 (Race 1), HG 2.5.7 (Race 5), HG 1.2.5.7 (Race 2), HG 0 (Race 3), and HG 1.3.6.7 (Race 14). The number of breeding lines in this panel is listed in **Table 1** and ranged from 150 lines for HG 1.3.6.7 (Race 14) to 1,136 lines for HG 0 (Race 3). SCN bioassays were performed for these lines over a period of 7 years from 2016 to 2022 at the Ashland Plant Growth Facility, University of Missouri-Columbia, MO, in accordance with the Standardized Cyst Evaluation Protocol (Niblack et al., 2009). Briefly, seedlings from each test line along with the susceptible controls (Lee 74 and Williams 82), seven indicator lines for the HG type test (Niblack et al., 2002), and Pickett, a differential line for SCN race test (Riggs and Schmitt, 1988), were transplanted into pots (100 cm^3^) of steam-pasteurized sandy loam soil. Each soybean line included five replicates organized in a randomized complete block design. Two days post-transplanting, seedlings were inoculated with 1,000 eggs, and the pots were suspended in temperature-controlled water tanks to maintain 27°C soil temperature. Twenty-eight days post-inoculation, the females from each sample were collected from the roots of each plant and manually counted using a stereo microscope. The mean number of cysts from each line was obtained, and a female index (FI) value was calculated by dividing the mean number of cysts from the test line by the mean number of cysts from the susceptible control and multiplying by 100. Lines with an extremely high number of females were not counted and assigned a FI value of ≥60.

**Table 1:** Summary table of QTNs identified for individual SCN populations in unfixed GWASs for Missouri and NUST panels along with average accuracies (%).

Panel	Statistical model	HG (race)	Number of genotypes	QTN SNP chromosome	QTN SNP position (Wm82.a2.v1)	QTN SNP ID	Average accuracy (%)	Candidate SCN resistance gene within 1 Mbp
**Missouri**	MLM	HG 2.5.7 (Race 1)	784	18	1,562,536	ss715629144	85%	*Glyma.18g022500*	
				11	32,959,788	ss715610417	75%	*Glyma.11g234500*	
				8	8,273,185	ss715602749	59%	*Glyma.08g108900*	
		HG 1.2.5.7 (Race 2)	801	18	1,562,536	ss715629144	82%	*Glyma.18g022500*	
				11	32,959,788	ss715610417	74%	*Glyma.11g234500*	
		HG 0 (Race 3)	1136	18	1,621,020	ss715629217	28%	*Glyma.18g022500*	
				8	8,273,185	ss715602749	53%	*Glyma.08g108900*	
		HG 2.5.7 (Race 5)	657	18	1,562,536	ss715629144	83%	*Glyma.18g022500*	
				11	32,959,788	ss715610417	73%	*Glyma.11g234500*	
				8	8,273,185	ss715602749	61%	*Glyma.08g108900*	
		HG 1.3.6.7 (Race 14)	150	18	1,621,020	ss715629217	33%	*Glyma.18g022500*	
	MLMM	HG 2.5.7 (Race 1)	784	18	1,562,536	ss715629144	85%	*Glyma.18g022500*	
				11	32,959,788	ss715610417	75%	*Glyma.11g234500*	
				8	7,959,982	ss715602729	58%	*Glyma.08g108900*	
		HG 1.2.5.7 (Race 2)	801	18	1,562,536	ss715629144	82%	*Glyma.18g022500*	
				11	32,959,788	ss715610417	74%	*Glyma.11g234500*	
				14	4,069,652	ss715618685	30%	*Glyma.14g054900*	
		HG 0 (Race 3)	1136	18	1,621,020	ss715629217	28%	*Glyma.18g022500*	
				8	8,273,185	ss715602749	54%	*Glyma.08g108900*	
		HG 2.5.7 (Race 5)	657	18	1,562,536	ss715629144	83%	*Glyma.18g022500*	
				11	32,959,788	ss715610417	73%	*Glyma.11g234500*	
				8	8,273,185	ss715602749	61%	*Glyma.08g108900*	
		HG 1.3.6.7 (Race 14)	150	18	1,621,020	ss715629217	33%	*Glyma.18g022500*	
**NUST**	MLM	HG 2.5.7 (Race 1)	512	18	1,562,536	ss715629144	61%	*Glyma.18g022500*	
		HG 0 (Race 3)	512	18	1,621,020	ss715629217	82%	*Glyma.18g022500*	
				7	36,493,756	ss715597434	27%	*Glyma.07g195900*	
	MLMM	HG 2.5.7 (Race 1)	512	18	1,562,536	ss715629144	61%	*Glyma.18g022500*	
		HG 0 (Race 3)	512	18	1,621,020	ss715629217	82%	*Glyma.18g022500*	
				6	9,163,989	ss715595659	48%	NA	
				14	45,991,145	ss715619181	51%	NA	

QTNs, quantitative trait nucleotides; SCN, soybean cyst nematode; GWAS, genome-wide association study; SNP, single-nucleotide polymorphism; MLM, Mixed Linear Model; MLMM, Multi-Locus Mixed-Model; NA, Not Applicable.

The second panel consisted of 512 breeding lines evaluated for SCN resistance within the USDA Uniform Soybean Tests—Northern Region (NUST panel) (Cai and Brock, 2022). USDA Uniform Soybean Tests—Northern Region aims to critically evaluate the soybean lines from the northern and mid-western states of the USA and provinces of Canada for yield, quality, and disease resistance. Lines within the NUST have been screened annually for SCN resistance at the University of Illinois SCN greenhouse since 2009. The SCN inbred populations HG 2.5.7 (Race 1) and HG 0 (Race 3) were used as inoculum, and a healthy seedling from each line was inoculated with 1,000 eggs from each SCN population separately. Three technical replicates for each line were infected and grown at a controlled soil temperature of 27°C for 30 days. Finally, the number of cysts was counted, and the phenotypes were reported as FI in the yearly NUST report.

### 2.2 Genotyping data and quality control

The genotypic data for the Missouri panel were assembled from the University of Missouri’s northern and southern breeding programs. Briefly, the genomic DNA for these lines was extracted with the cetyl trimethyl ammonium bromide (CTAB) method (Doyle and Doyle, 1987), and SNP genotyping was performed at Soybean Genomics and Improvement Laboratory, USDA-ARS, Beltsville, MD, using Illumina Infinium BARCSoySNP6K BeadChip (Song et al., 2020). From over 6,000 resulting SNPs, a total of 4,383 high-quality SNPs were selected for the analysis after eliminating the SNPs with more than 10% of missing data or heterozygosity and minor allele frequencies of less than 5%. For the NUST panel, the Illumina array-based SNP genotyping data (SoySNP6K) for cultivars were obtained from the SoybeanBase website (https://soybeanbase.breedinginsight.net/), and a total of 4,315 high-quality SNPs were obtained after quality control using the same criteria as previously described.

### 2.3 Genome-wide association study for resistance to SCN

Initially, GWASs were performed for individual SCN populations in each of the full GWAS panels. For this, both Mixed Linear Model (MLM) (Yu et al., 2006) and Multi-Locus Mixed-Model (MLMM) (Segura et al., 2012) algorithms were used and implemented in R statistical software using the Genomic Association and Prediction Integrated Tool (GAPIT) package (Wang and Zhang, 2021). These analyses are referred to as unfixed GWASs in this study. The optimal number of principal components (PCs) was identified visually from the screen plots, and a kinship matrix was calculated using the VanRaden method (VanRaden, 2008). The resulting population structure (Q Matrix) and kinship matrix (K Matrix) were incorporated into the statistical models as covariates to account for false positives (Wang and Zhang, 2021).

After the initial unfixed GWASs, each panel was fixed separately for the alternate allele of the chromosome 18 QTNs detected in individual SCN population GWASs by eliminating all the individuals carrying the Williams 82 version of this QTN in each panel. Consequently, the Missouri panel was fixed for the alternate allele of QTN18a (ss715629144 SNP) for HG 2.5.7 (Race 1), HG 1.2.5.7 (Race 2), and HG 2.5.7 (Race 5), while for HG 0 (Race 3) and HG 1.3.6.7 (Race 14), the panel was fixed for the alternate allele of QTN18b (ss715629217 SNP). Similarly, the NUST panel was fixed for the alternate allele of QTN18a for HG 2.5.7 (Race 1) and the alternate allele of QTN18b for HG 0 (Race 3). After the creation of each new panel, all the GWASs were conducted again using only the MLMM method with the same criteria as defined for the unfixed GWASs. These subsequent GWASs are referred to as fixed GWASs from here onward.

The average accuracy values for individual SNPs in each GWAS were computed using the Accuracy concept (Škrabišová et al., 2022), which enables the assessment of direct correspondence between a phenotype and a genotype and can be implemented using the AccuCalc package (Biová et al., 2023). Since the Accuracy concept can be utilized only for the binomial phenotypes, the SCN virulence phenotypes were converted based on the female index into a binary format where the lines with FI ≤ 30 were assigned a mutant (MUT) phenotype while the lines with FI ≥ 30 were assigned wild-type (WT) phenotypes. The average accuracy (Avr_acc) for individual SNPs was computed according to the following equation devised by Škrabišová and colleagues:


$$Average\;Accuracy(\%)=(\frac{WT\;Accuracy+Mutant\;Accuracy}{2})\times 100$$

This accuracy measure was integrated into the individual Manhattan plots to illustrate the measure of direct correspondence between the SNP markers and the binary phenotypes. Further, reported SCN resistance or *α-SNAP* genes within 1 Mbp from the QTNs were included in the plots along with their distance from the QTNs.

### 2.4 Evaluation of SCN phenotypes by allelic combinations

To evaluate the differences in mean FIs for Missouri panel lines carrying different allelic combinations of QTNs detected in individual population GWAS, the female indices for genotypes were plotted by allelic combinations. Separate plots were created for QTNs detected in fixed and unfixed GWASs. The *Rhg1* genotypes derived from two KASP assays (Rhg1-2 and SNAP18-1) were used to distinguish between reference susceptible allele *Rhg1-c* and two resistance alleles *rhg1-a* and *rhg1-b* and overlaid on the resulting plots (Kadam et al., 2016; Usovsky et al., 2021).

### 2.5 *Rhg* alleles and accuracy analysis

To confirm variant positions of previously proposed causal mutations (CMs) and to analyze additional alleles at the different *Rhg* loci, we explored allelic variation by utilizing the Soybean Allele Catalog (Chan et al., 2023). Here, we queried *Rhg1*/*GmSNAP18* (*Glyma.18g022500*), *Rhg4*/*GmSHMT08*, and *Rhg2* (*Glyma.11g234500*) and visualized the results.

Accuracy analysis serves the purpose of direct correspondence assessment between a genotype and a phenotype and a binary phenotype and a variant position (Škrabišová et al., 2022). Briefly, it is based on the fact that many genetic variants are of a biallelic nature. Therefore, if an observed phenotype is categorized into a binomial distribution (WT/MUT), Accuracy can be calculated for every variant position in the genotype. Therefore, in forward GWASs, Accuracy serves as a measure of direct correspondence between a binarized phenotype and a marker position derived from low-density genotyping data. In forward GWASs, Accuracy explores the correspondence between an observed phenotype and variant positions (markers, CM, or other variant positions), whereas in inverse GWASs, the landscape of association with the CM is revealed if the gene has been cloned and CM is known. In inverse GWASs, the CM variant position is used as a synthetic phenotype (Škrabišová et al., 2022). Here, we used Accuracy in both GWAS directions. By inverse GWASs, we aimed to evaluate how well the associated markers of the SoySNP6K chip can predict the individual CMs. In forward GWASs, we ascertained the correspondence of individual CMs and other associated variant positions to resistance against individual SCN populations.

First, we performed the inverse GWAS with the Soy775 whole-genome sequencing (WGS) data set in the AccuTool (https://soykb.org/AccuTool/index.php) (Škrabišová et al., 2022) by using the following candidate CMs: *rhg1-a* (Gm18:1,643,660 Wm.82.a2.; allele D208E), *rhg1-b* (Gm18:1,643,643; allele Q203K), *Rhg4-a* (Gm08:8,361,148; allele P200R) (Patil et al., 2019), and previously identified splice site position at the *Rhg2* (Gm11:32,969,916; splice) (Lakhssassi et al., 2017; Bayless et al., 2018; Shaibu et al., 2022). We used CMs as synthetic phenotypes (reference base at the CM position as WT phenotype and alternate base as MUT phenotype) individually and defined an arbitrary range of plus and minus 2 Mbp around the CM for the inverse GWASs. We selected the option to return only SoySNP50K positions and sorted the results for descending Average accuracy.

For Accuracy analysis, we utilized a subset of 1,066 whole genome resequenced (WGRS) soybean accessions (Soy1066 data panel) available at SoyKB (https://soykb.org/). This panel contains publicly available data sets and has been filtered to contain approximately 38 million high-quality polymorphic positions (Chan et al., 2023). The panel was subsetted to include the lines for which SCN female indices were reported by Patil et al. (2019). Two categories of SCN resistance status as a binarized phenotype (WT or MUT) were used for this analysis, and all the lines with FI > 60 were coded as SCN-susceptible and therefore assigned as WT. In contrast to WT accessions, we categorized only those accessions as MUT that exhibited resistance to SCN with FI< 30 for the *rhg1* and *Rhg2* calculations or FI< 10 for the *Rhg4* calculation. To maximize the statistical power of the Accuracy analysis, we increased the number of WT accessions by adding those of Soy1066 with susceptible SCN resistance status according to phenotypes publicly available in the GRIN database (https://www.ars-grin.gov; downloadable at Soybase, https://soybase.org/grindata/). For this, we considered an accession as being susceptible (WT) if it had susceptible phenotypes for all SCN populations available in the GRIN. In total, there were 135 accessions in the Soy1066 that we coded as WT/SCN-susceptible phenotype. For each SCN resistance locus, a 10-Mbp genomic region around the tagging markers was analyzed by the AccuCalc tool to ascertain accuracy (Biová et al., 2023).

Finally, to dissect the effect of individual *Rhg* alleles on resistance to SCN populations, we performed the Accuracy analysis independently for HG 2.5.7 (Race 1) and HG 0 (Race 3). For this purpose, we subsetted the accessions based on their *rhg1-a*, *rhg1-b*, and *Rhg4* allele status and calculated Accuracy for every variant position in the 10-Mbp regions by AccuCalc. Further, we processed the results as follows: we filtered the variant positions for WT accuracy (Acc_WT) ≥90.00%, sorted the variant positions by descending Average accuracy, and further subsetted the results for functional effect predicted variant positions only (moderate and high SNPEff (Cingolani et al., 2012) effect classes).

## 3 Results

### 3.1 Genome-wide marker associations for SCN resistance in Missouri panel

**Figure 1:**
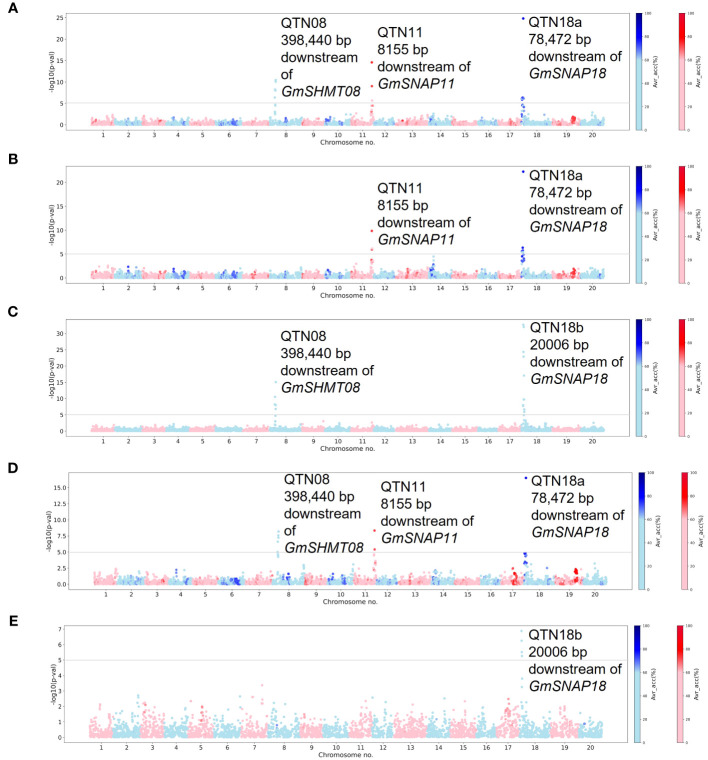
Manhattan plots generated from genome-wide association study (GWAS) analyses for soybean cyst nematode (SCN) resistance within the Missouri panel using Mixed Linear Model (MLM). The Manhattan plots highlight the average accuracy calculations (using a color scale on the right) for individual single-nucleotide polymorphisms (SNPs), calculated using the AccuCalc package. **(A)** Manhattan plot for HG 2.5.7 (Race 1). **(B)** Manhattan plot for HG 1.2.5.7 (Race 2). **(C)** Manhattan plot for HG 0 (Race 3). **(D)** Manhattan plot for HG 2.5.7 (Race 5). **(E)** Manhattan plot for HG 1.3.6.7 (Race 14).

GWASs were performed for individual SCN populations using MLM and MLMM statistical models in the individual curated panels of breeding lines (Missouri and NUST panels). Here, we used both MLM and MLMM statistical models to determine the model with higher statistical power and accuracy. The QTNs detected in each of these analyses are reported in **Table 1**. A total of four significant QTNs on chromosomes 8, 11, and 18 (QTN08, QTN11, QTN18a, and QTN18b) were detected as significantly associated (−log10 (p-val) >5) with SCN resistance using the MLM statistical model (**Figure 1**). For HG 2.5.7 (Race 1) and HG 2.5.7 (Race 5) populations, three identical QTNs were detected on chromosomes 8, 11, and 18 (QTN08, QTN11, and QTN18a) (**Figures 1A, D**). Identical QTNs were detected on chromosomes 11 and 18 (QTN11 and QTN18a) for resistance to HG 1.2.5.7 population (**Figure 1B**); however, no QTN was detected on chromosome 8. A QTN on chromosome 18 (QTN18b) was also detected for HG 0 (**Figure 1C**) and HG 1.3.6.7 (**Figure 1E**), which was ~60,000 bp upstream from QTN18a identified for other HG types. Additionally, QTN08 was identified for HG 0; however, no further QTNs were detected for HG 1.3.6.7.

A total of six significant QTNs were detected on chromosomes 8, 11, 14, 17, and 18 (QTN08, QTN11, QTN14, QTN17, QTN18a, and QTN18b) within the Missouri panel (**Supplementary Figure 1**) using the MLMM statistical model. Additional QTNs on chromosomes 14 and 17 were detected for HG 1.2.5.7 and HG 2.5.7 (Race 5), respectively (**Supplementary Figures 1B, D**). The average accuracy values for the QTN11 and QTN18a were above 70% for HG 2.5.7 (Race 1), HG 1.2.5.7 (Race 2), and HG 2.5.7 (Race 5), indicating high direct correspondence between associated markers and the phenotype; however, the accuracy values were approximately 50% for QTN08, indicating lower correspondence. The average accuracy values of QTNs detected for resistance to HG 0 (Race 3) and HG 1.3.6.7 (Race 14) were approximately 50% or below, indicating the low direct correspondence between associated markers and phenotypes. QTN08, QTN11, QTN14, and QTN18a/QTN18b identified in these analyses were within a 1-Mbp genomic region surrounding the annotated genes *GmSHMT08*, *GmSNAP11*, *GmSNAP14*, and *GmSNAP18*, respectively, while no such known SCN resistance genes were identified within a 1-Mbp region surrounding QTN17.

### 3.2 Genome-wide marker associations for SCN resistance in NUST panel

**Figure 2:**
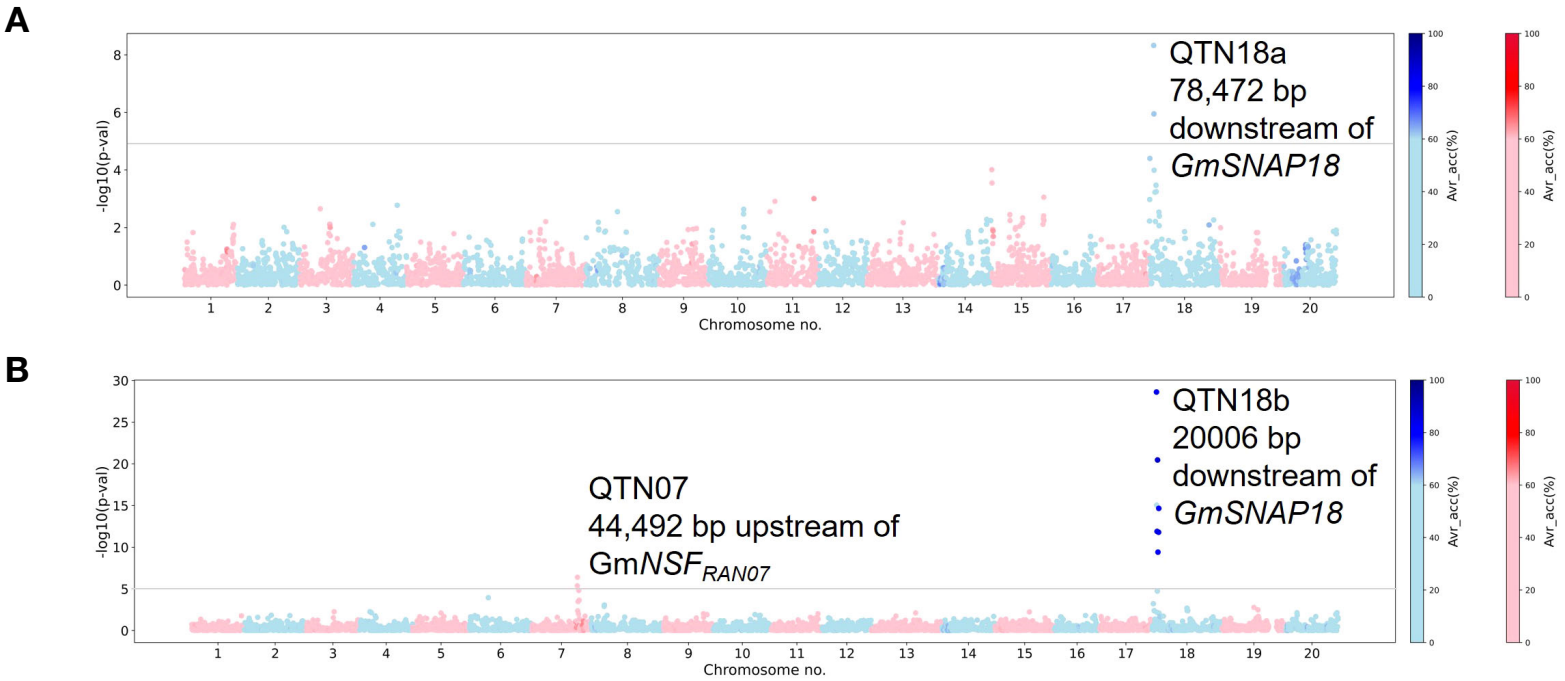
Manhattan plots generated from genome-wide association study (GWAS) analyses for soybean cyst nematode (SCN) resistance within the NUST panel using Mixed Linear Model (MLM). The Manhattan plots highlight the average accuracy calculations (using a color scale on the right) for individual single-nucleotide polymorphisms (SNPs), calculated using the AccuCalc package. **(A)** Manhattan plot for HG 2.5.7 (Race 1). **(B)** Manhattan plot for HG 0 (Race 3).

In total, five loci on chromosomes 6, 7, 14, and 18 (QTN06, QTN07, QTN14n, QTN18a, and QTN18b) were detected as significantly associated (−log10 (p-val) >5) with SCN resistance within the NUST panel. For HG 2.5.7 (Race 1), QTN18a was detected using both MLM and MLMM methods (**Figure 2A**, **Supplementary Figure 2A**). A QTN on chromosome 18 (QTN18b) was detected for HG 0 along with a QTN on chromosome 7 (QTN07) using MLM analysis (**Figure 2B**). In addition to the QTN18b, QTNs on chromosomes 6 and 14 were detected for HG 0 (Race 3) when using the MLMM model; however, the QTN07 was not detected (**Supplementary Figure 2B**). The average accuracy value for QTN18a and QTN18b in this analysis was above 60%, while low average accuracies were observed for other QTNs identified in this panel. The QTN07, QTN18a, and QTN18b identified in these analyses were within the 1-Mbp genomic region surrounding the reported *GmNSF_RAN07_* and *GmSNAP18* genes, while no genes related to SCN resistance have been identified in the 1-Mbp genomic region surrounding QTN06 and QTN14n. Due to the discrepancy of QTN06 and QTN14n between MLM and MLMM models and the lack of reported SCN resistance genes in the surrounding regions, these QTNs were considered spurious associations in this analysis.

### 3.3 Fixed-effect genome-wide marker associations for minor effect QTNs contributing to SCN resistance

After the initial GWASs using the Missouri and NUST panels, we fixed both panels for the alternate allele of the chromosome 18 QTNs (QTN18a or QTN18b) detected in each SCN population GWAS. This was achieved by discarding lines that carried the reference (Williams 82) version of these QTNs from the unfixed panel. Using these fixed-effect panels, we performed GWASs to identify minor effect loci relative to the large effects of the *rhg1-a* and *rhg1-b* alleles. We utilized MLMM for this analysis due to its superiority in statistical power over MLM. The total number of genotypes in each analysis and the peak significant SNPs are reported in **Table 2**.

**Table 2:** Summary table of QTNs identified for individual SCN populations in fixed GWASs for Missouri and NUST panels along with average accuracies (%).

Panel	Statistical model	HG (race)	Number of genotypes	QTN SNP chromosome	QTN SNP position (Wm82.a2.v1)	QTN SNP ID	Average accuracy (%)	Candidate SCN resistance gene within 1 Mbp
**Missouri**	MLMM	HG 2.5.7 (Race 1)	306	11	32,959,788	ss715610417	23%	*Glyma.11g234500*
				8	8,273,185	ss715602749	56%	*Glyma.08g108900*
				14	4,100,480	ss715618699	34%	*Glyma.14g054900*
				17	13,176,053	ss715626052	67%	NA
		HG 1.2.5.7 (Race 2)	311	11	32,959,788	ss715610417	27%	*Glyma.11g234500*
				14	4,100,480	ss715618699	22%	*Glyma.14g054900*
				2	44,427,664	ss715583112	52%	*Glyma.02g260400*
		HG 0 (Race 3)	273	8	8,273,185	ss715602749	56%	*Glyma.08g108900*
				18	2,229,173	ss715629936	25%	*Glyma.18g022500*
				11	32,959,788	ss715610417	47%	*Glyma.11g234500*
				17	18,790,751	ss715626347	46%	NA
		HG 2.5.7 (Race 5)	263	11	32,959,788	ss715610417	25%	*Glyma.11g234500*
				8	8,273,185	ss715602749	59%	*Glyma.08g108900*
				17	13,176,053	ss715626052	75%	NA
				14	4,856,342	ss715619352	42%	*Glyma.14g054900*
**NUST**	MLMM	HG 2.5.7 (Race 1)	481	18	1,621,020	ss715629217	63%	*Glyma.18g022500*
		HG 0 (Race 3)	128	18	665,442	ss715632544	25%	*Glyma.18g022500*
				20	44,272,285	ss715638506	38%	NA

QTNs, quantitative trait nucleotides; SCN, soybean cyst nematode; GWAS, genome-wide association study; SNP, single-nucleotide polymorphism; MLM, Mixed Linear Model; MLMM, Multi-Locus Mixed-Model; NA, Not Applicable.

**Figure 3:**
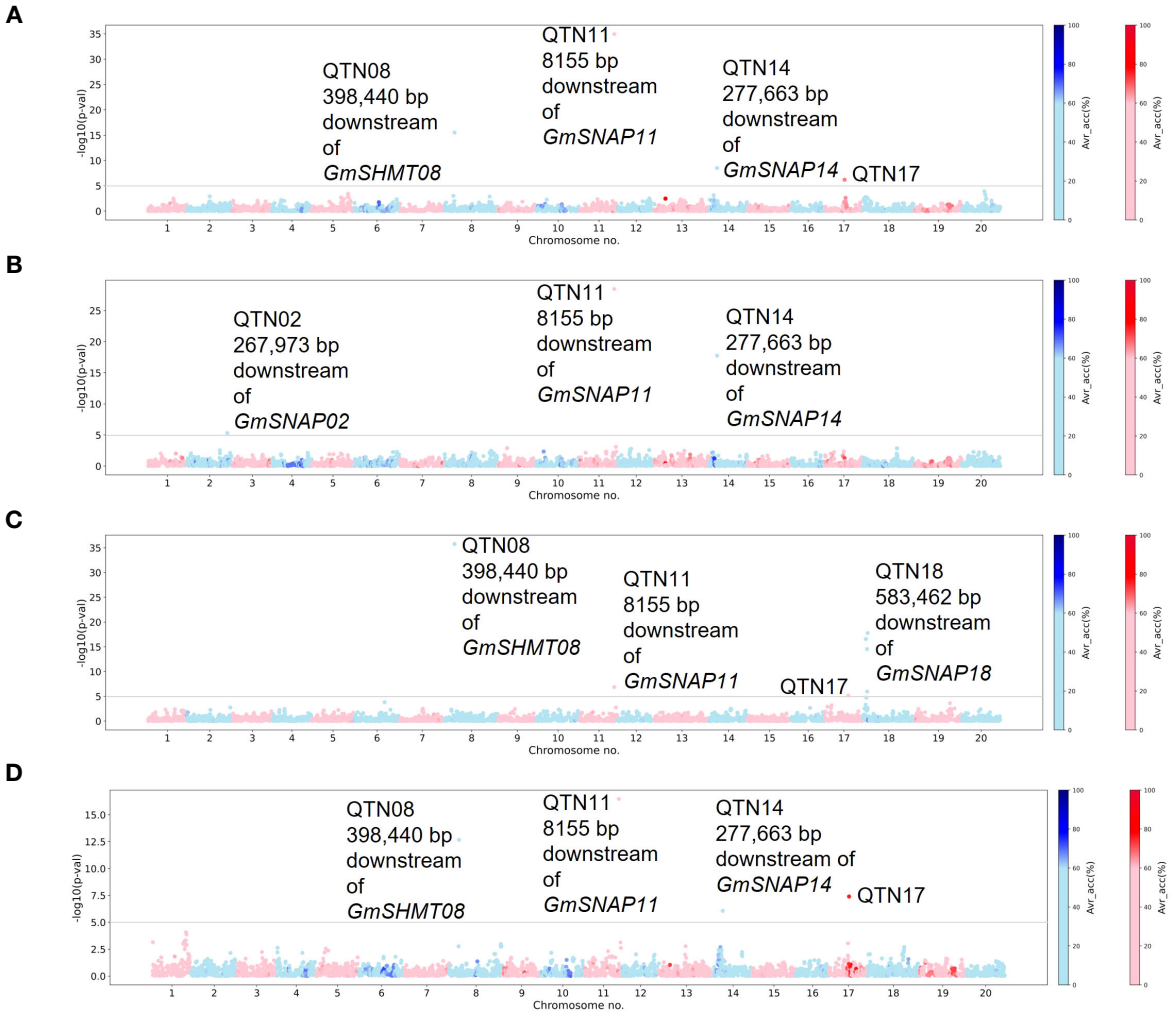
Manhattan plots generated from Multi-Locus Mixed-Model (MLMM)-based genome-wide association study (GWAS) analyses for soybean cyst nematode (SCN) resistance within Missouri panel fixed for the alternate allele of the chromosome 18 quantitative trait nucleotide (QTN) detected in individual SCN race unfixed GWASs. The lines were fixed for the alternate allele of QTN18a (ss715629144) for HG 2.5.7 (Race 1), HG 1.2.5.7 (Race 2), and HG 2.5.7 (Race 5), while for HG 0 (Race 3), the panel was fixed for the alternate allele of QTN18b (ss715629217). The Manhattan plots highlight the average accuracy calculations (using a color scale on the right) for individual single-nucleotide polymorphisms (SNPs), calculated using the AccuCalc package. **(A)** Manhattan plot for HG 2.5.7 (Race 1). **(B)** Manhattan plot for HG 1.2.5.7 (Race 2). **(C)** Manhattan plot for HG 0 (Race 3). **(D)** Manhattan plot for HG 2.5.7 (Race 5).

For the fixed Missouri panel, QTN17 was detected for HG 2.5.7 (Race 1) in addition to HG 2.5.7 (Race 5) (**Figure 3**). A QTN on chromosome 17 (QTN17) was also detected for HG 0; however, this QTN was located 1.6 Mbp upstream of QTN17 detected for HG 2.5.7 (Race 1) and HG 2.5.7 (Race 5). An additional QTN on chromosome 2 was identified for HG 1.2.5.7. Furthermore, the QTN14 detected for HG 1.2.5.7 in the unfixed GWAS was also detected for HG 2.5.7 (Race 1 and 5) in this analysis. The chromosome 2 QTN identified in this analysis was within the 1-Mbp genomic region surrounding the annotated *GmSNAP02* gene. The chromosome 17 QTN average accuracy value in this analysis was above 70%, while low average accuracies were observed for all other QTNs identified in this panel.

For the fixed NUST panel, no additional QTNs were detected for HG 2.5.7 (Race 1). However, for HG 0, the previously detected QTNs on chromosomes 6, 7, and 14 were not detected in this analysis, while an additional QTN on chromosome 20 was detected (**Supplementary Figure 3B**). No reported SCN resistance genes were located within a 1-Mbp region surrounding the chromosome 20 QTN, and the average accuracy value for all the QTNs identified in this panel was low. The chromosome 20 QTN identified in the fixed NUST GWASs was not analyzed further in our study due to inconsistency across different methodologies and a lack of reported SCN resistance genes in the region.

### 3.4 Impact of allelic combinations on different SCN populations in Missouri panel

To elucidate the complex genetic architecture of resistance to different SCN populations, we plotted the allelic combinations of the observed QTNs in the Missouri panel against their FIs. Further, we added the information from KASP marker data to distinguish between the effect of *Rhg1* resistance alleles (*rhg1-a* and *rhg1-b*). This analysis was not performed for the NUST panel due to the lack of availability of the KASP marker data for that panel.

**Figure 4:**
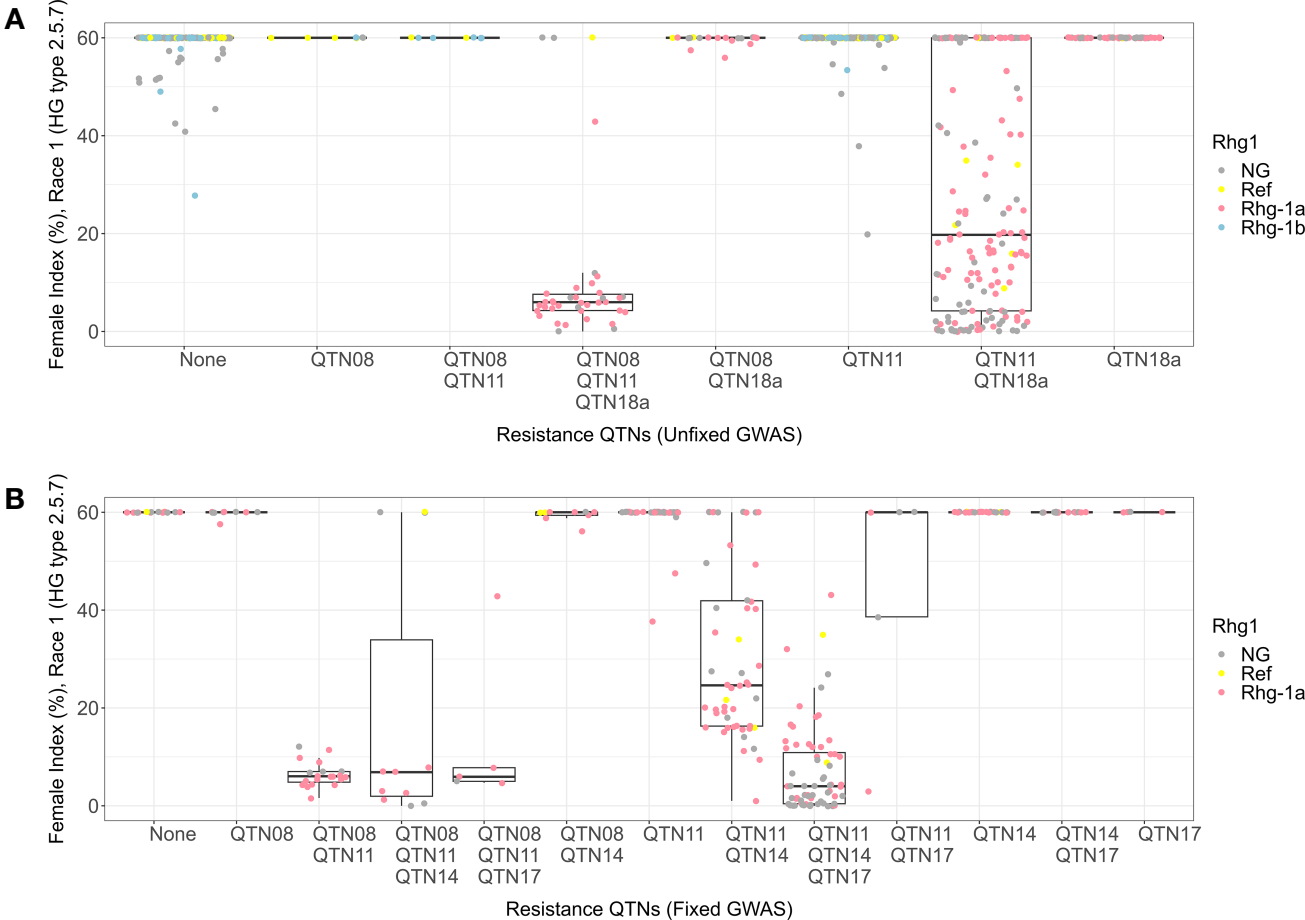
Female index score for lines in Missouri panel to HG 2.5.7 (Race 1) plotted by resistance quantitative trait nucleotides (QTNs). **(A)** Grouped by resistance QTNs identified in unfixed genome-wide association study (GWAS). **(B)** Grouped by resistance QTNs identified in GWAS with genotypes fixed for QTN18a (ss715629144). The x-axis represents resistance QTNs in the breeding lines, while the y-axis represents female indices (FI %). The data points are colored for different alleles of *Rhg1* observed using KASP assays according to the color scheme in the legend. NG in the legend stands for “not genotyped”.

For HG 2.5.7 (Race 1), among the allelic combinations for the QTN08, QTN11, and QTN18a detected in the unfixed GWAS, the combination containing alternate alleles for all three QTNs was the most resistant genotype (median FI = 7) (**Figure 4A**). The combination with reference allele for QTN08 and alternate alleles for other detected QTNs was the second most resistant genotype among all combinations (median FI = 20) and had a larger phenotypic range than the three alternate allele combinations. Furthermore, the *Rhg1* allele genotypes from KASP markers indicated that *rhg1-a* allele is necessary for resistance in the allelic combinations described above, while *rhg1-b* does not contribute to resistance in identical combinations with QTN08 and QTN11. All the other combinations showed susceptible phenotypes regardless of the allelic status of the QTN18a.

When we fixed the Missouri panel for the alternate allele of QTN18a, KASP marker data showed that the remaining lines contained only the reference and *rhg1-a* alleles of *Rhg1*. The absence of *rhg1-b* alleles from the fixed panel highlights the accuracy of the QTN18a in distinguishing between *rhg1-b* and *rhg1-a* alleles within this panel of soybean lines (**Figure 4B**). The allelic combinations of QTNs detected in the HG 2.5.7 fixed GWAS panel indicated that the combinations with alternate alleles of QTN08 and QTN11 were resistant to HG 2.5.7 (Race 1) in the presence of the *rhg1-a* allele. Furthermore, it was observed that the alternate alleles for QTN17 and QTN14 imparted resistance (median FI = 2) in combination with *rhg1-a* and *rhg2* when the reference allele was present for QTN08. Only moderate resistance was observed when the reference allele of QTN17 was present in such a combination.

**Figure 5:**
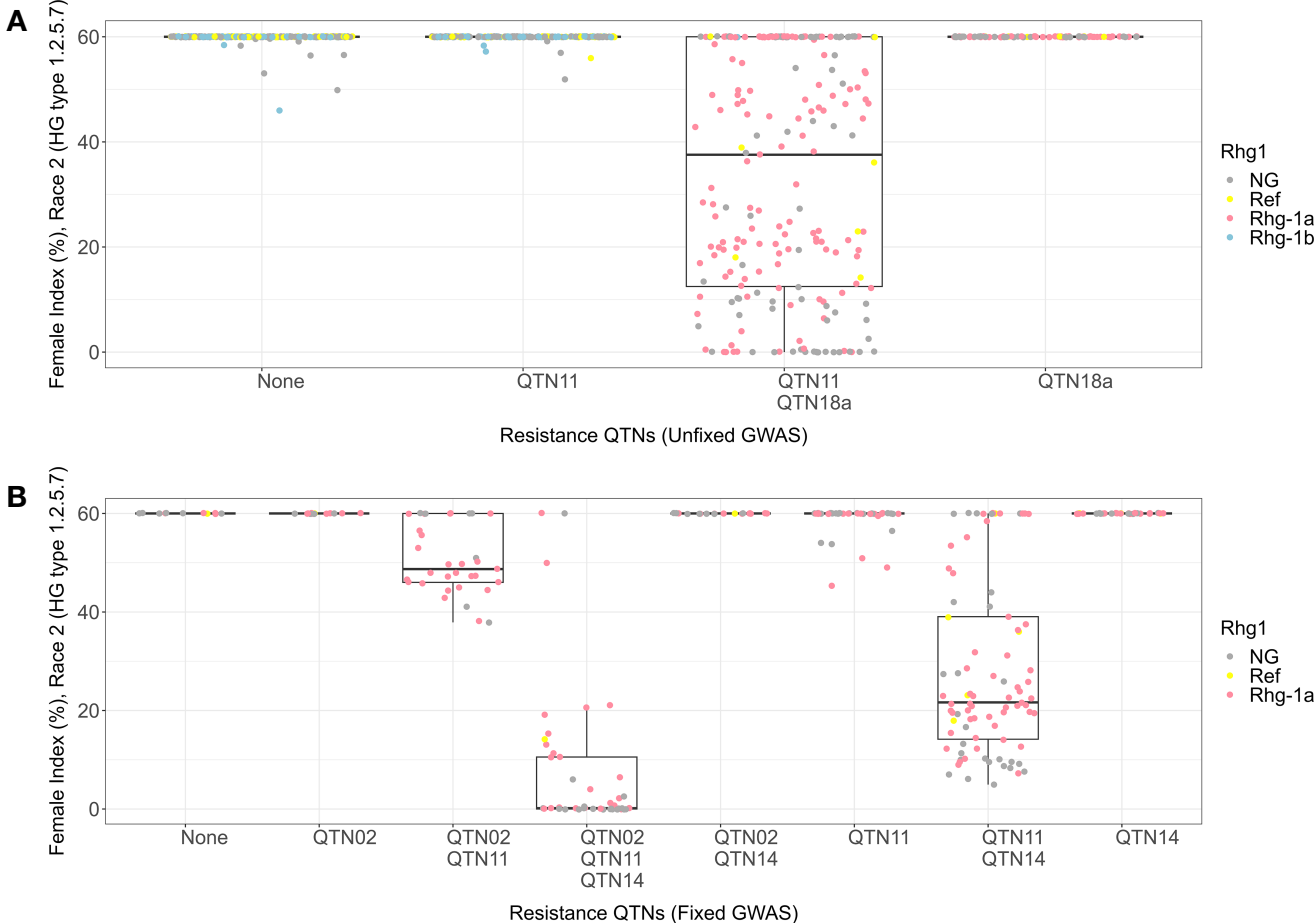
Female index score for lines in Missouri panel to HG 1.2.5.7 (Race 2) plotted by resistance quantitative trait nucleotides (QTNs). **(A)** Grouped by resistance QTNs identified in unfixed genome-wide association study (GWAS). **(B)** Grouped by resistance QTNs identified in GWAS with genotypes fixed for QTN18a (ss715629144). The x-axis represents resistance QTNs in the breeding lines, while the y-axis represents female indices (FI %). The data points are colored for different alleles of *Rhg1* observed using KASP assays according to the color scheme in the legend. NG in the legend stands for “not genotyped”.

For HG 1.2.5.7, the combination with alternate alleles for QTN11 and QTN18a detected in unfixed GWAS exhibited a wide range of phenotypes ranging from susceptible to resistant (**Figure 5A**). Genotypes from KASP marker analysis indicated that most lines in this combination carried the *rhg1-a* allele. All the other combinations in this analysis exhibited susceptible phenotypes. Fixing the panel for alternate allele of QTN18a resulted in lines containing only the reference and *rhg1-a* alleles of *Rhg1*. Among the allelic combinations of the QTN02, QTN11, and QTN14 in the HG 1.2.5.7 fixed GWAS (**Figure 5B**), the combination with alternate alleles of QTN11 and QTN14 and reference allele of QTN02 showed resistant phenotype (median FI = 0), while the combination of the three alternate alleles together showed a moderately resistant phenotype with a larger range of female indices among those lines (median FI = 21). Combined with genotypes from KASP markers, this result indicates that *rhg1-a* with the alternate alleles QTN11 and QTN14 conferred resistance to HG 1.2.5.7.

**Figure 6:**
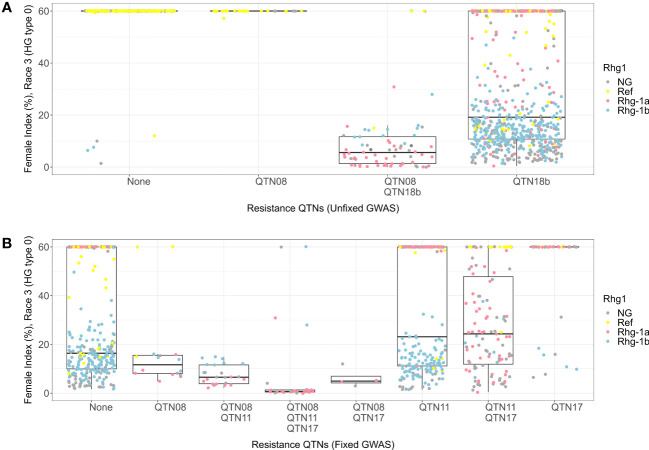
Female index score for lines in Missouri panel to HG 0 (Race 3) plotted by resistance quantitative trait nucleotides (QTNs). **(A)** Grouped by resistance QTNs identified in unfixed genome-wide association study (GWAS). **(B)** Grouped by resistance QTNs identified in GWAS with genotypes fixed for QTN18b (ss715629217). The x-axis represents resistance QTNs in the breeding lines, while the y-axis represents female indices (FI %). The data points are colored for different alleles of *Rhg1* observed using KASP assays according to the color scheme in the legend. NG in the legend stands for “not genotyped”.

The lines with alternate alleles of QTN08 and QTN18b detected in HG 0 unfixed GWAS demonstrated resistance (median FI = 5) (**Figure 6A**). The combination with the reference allele of QTN08 and alternate allele QTN18b also showed resistance (median FI = 19); however, the spread of FIs among lines was much wider than the combination with alternate alleles of both QTNs. Further, from the KASP marker genotypes, it was observed that both *rhg1-a* and *rhg1-b* alleles were involved in resistance to HG 0 and that the QTN18b detected here was unable to distinguish between these alleles. The allelic combinations of QTN08, QTN11, and QTN17 detected for the HG 0 using fixed GWAS indicated that the combination of alternate alleles of these QTNs had the lowest median FI (**Figure 6B**). The KASP marker data revealed this allelic combination in the presence of *rhg1-a* conferred high resistance to HG 0 (median FI = 0). Further, it was observed that the *rhg1-b* allele conferred resistance to HG 0 regardless of the allelic status of other QTNs.

**Figure 7:**
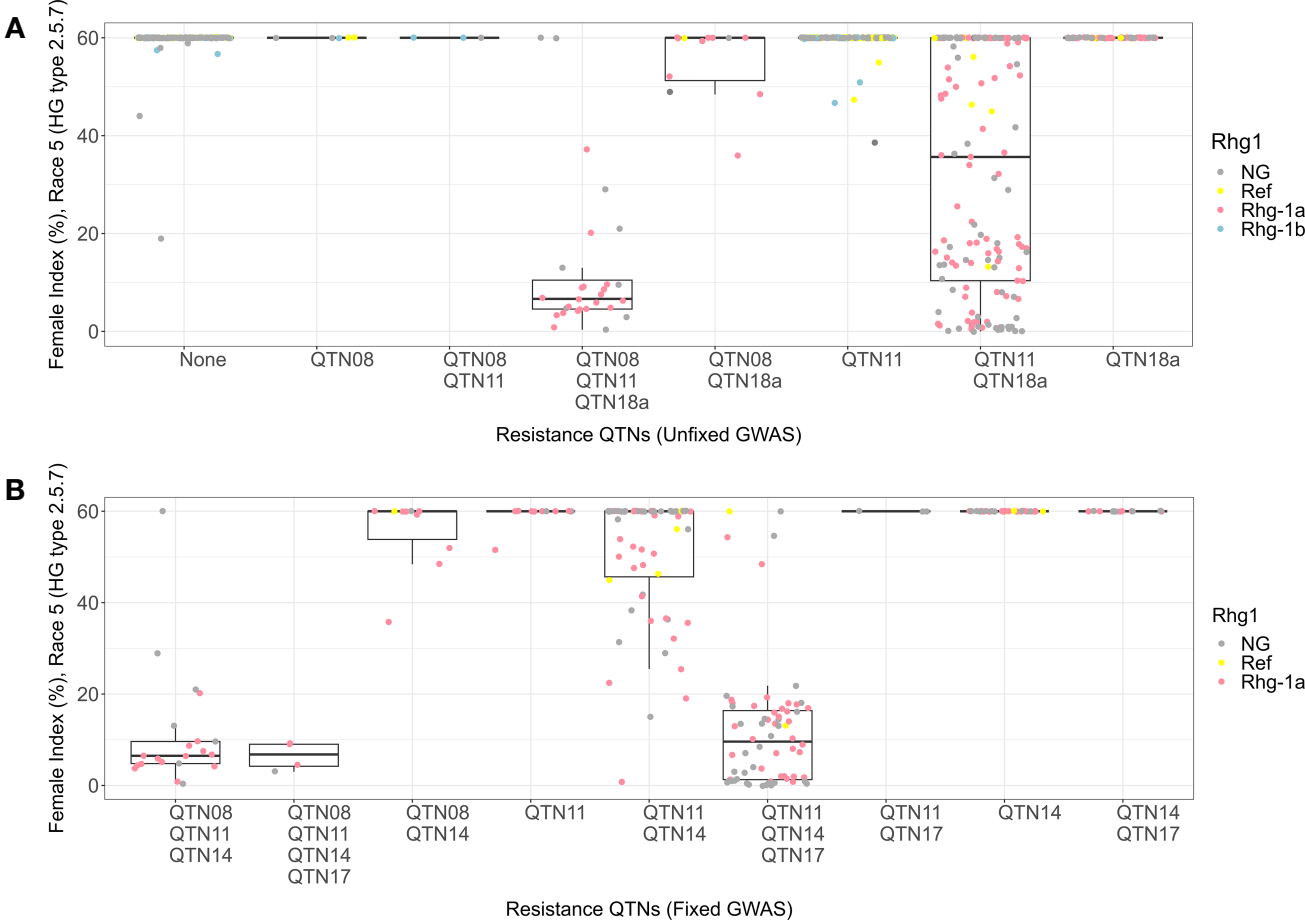
Female index score for lines in Missouri panel to HG 2.5.7 (Race 5) plotted by resistance quantitative trait nucleotides (QTNs). **(A)** Grouped by resistance QTNs identified in unfixed genome-wide association study (GWAS). **(B)** Grouped by resistance QTNs identified in GWAS with genotypes fixed for QTN18a (ss715629144). The x-axis represents resistance QTNs in the breeding lines, while the y-axis represents female indices (FI %). The data points are colored for different alleles of *Rhg1* observed using KASP assays according to the color scheme in the legend. NG in the legend stands for “not genotyped”.

For HG 2.5.7 (Race 5), the allelic combination with alternate alleles for QTN08, QTN11, and QTN18a detected in HG 2.5.7 (Race 5) unfixed GWAS had a resistant phenotype with median FI equal to 6 (**Figure 7A**). The combination with the reference allele of QTN08 and alternate alleles of QTN11 and QTN18a displayed a moderately susceptible phenotype (median FI = 36) with a wide distribution of FIs. With insights from KASP marker data, this indicates that *rhg1-a* along with alternate alleles of QTN08 and QTN11 conferred resistance to HG 2.5.7 (Race 5). When we fixed the Missouri panel for the alternate allele of QTN18a, the KASP marker data showed that the remaining lines only contained reference and *rhg1-a* alleles of *Rhg1*. Among the allelic combinations for QTN08, QTN11, QTN14, and QTN17 detected in HG 2.5.7 (Race 5) fixed GWAS, the combinations with alternate alleles of QTN08 and QTN11 demonstrated resistant phenotypes (median FI = 7) (**Figure 7B**). Additionally, it was observed that the alternate alleles of QTN14 and QTN17 imparted resistance when the reference allele of QTN08 and the alternate allele of QTN11 were present. The remaining combinations in this analysis were not effective and exhibited susceptible phenotypes.

### 3.5 Accuracy of GWAS QTN to the causal mutations

To analyze previously identified alleles of SCN resistance genes in the context of a diverse panel of 1,066 soybean resequenced accessions, we queried *Rhg* loci in the Soybean Allele Catalog. Among the alleles, we identified variant positions of the known causal and candidate mutations. For *Rhg1* (*Glyma.18g022500*), we detected the resistance alleles *rhg1-a* (Gm18:1,643,660 Wm82.a2; allele D208E) and *rhg1-b* (Gm18: 1, 643, 643; allele Q203K). For *Rhg4* (*Glyma.08g108900*), we identified the resistance allele at position (Gm08:8,361,148; allele P200R). For *Rhg2* (*Glyma.11G234500*), we detected the resistance allele *rhg2* at the position Gm11:32,969,916 that causes mis-splicing resulting in a premature stop codon (Lakhssassi et al., 2017; Bayless et al., 2018). We used these positions in subsequent Accuracy analyses.

By inverse GWAS, we aimed to evaluate how well the associated markers of the SoySNP6K chip can predict the individual CMs. To assess the direct correspondence between the individual CMs and the low-density markers, we calculated the Accuracy of each of the CMs that were used as a synthetic phenotype (Škrabišová et al., 2022). Here, we used variant positions of the identified CMs for *rhg1-a*, *rhg1-b*, *Rgh4* (Patil et al., 2019), and *rhg2* (Matsye et al., 2012) in inverse GWASs with SoySNP6K chip low-density data as the genotype. The results revealed 85% average accuracy (Avg_Accu) for *rhg1-a* (D208E) to QTN18a detected for HG 2.5.7 (Races 1 and 5) and HG 1.2.5.7 (Race 2). For the *rhg1-b* (Q203K) inverse analysis, QTN18b detected for HG 0 (Race 3) and HG 1.3.6.7 (Race 14) had 76% Avg_Accu, while QTN18a had less specificity (67% Avg_Accu). The *Rhg4* (P200R) Avg_Accu to the QTN08 was 94%, and the *rhg2* (splice) Avg_Accu to QTN11 was 83%. This analysis demonstrated that QTN18a could be used as a proxy for *rhg1-a*, while QTN18b could be used to a lesser extent for *rhg1-b*. The QTN08 had the highest accuracy for detection of resistance from *Rhg4*, and QTN11 could be used as a proxy for *rhg2*.

The observed phenotype accuracy analysis was performed with the Soy1066 WGS data set with available SCN resistance phenotype information: a subset of 135 fully SCN susceptible accessions and up to 28 accessions with SCN resistance (FI< 30) specific to HG 2.5.7 (Races 1 and 5), HG 1.2.5.7 (Race 2), HG 0 (Race 3), and/or HG 1.3.6.7 (Race 14). Although this analysis had low power due to the limited number of resistant accessions, the analysis prioritizing the susceptible (WT) accessions was able to dissect the *rhg1-a* and *rhg1-b* alleles and confirm the connection between the GWAS QTNs on chromosomes 11 and 8 with *rhg2* and *Rhg4*, respectively. The results demonstrated that QTN18a more specifically predicted the *rhg1-a* allele compared to *rhg1-b*, which is in accordance with the higher frequency of *rhg1-a* allele in the indicator lines and thus also represents the majority of SCN-resistant lines of the Soy1066 data set (**Supplementary Table 1**) (Patil et al., 2019). For highly resistant accessions (FI = 0 to 9) to SCN population HG 2.5.7 (Race 1), the *Rhg4* tagging QTN08 had high accuracies (93% Avg_Accu) with the SCN resistance phenotype (**Supplementary Table 1**). For accessions resistant to HG 2.5.7 (Race 1) and HG 0, the accuracies were 89% and 87% for the *rhg2* tagging QTN11, respectively.

Another observed phenotype Accuracy analysis was performed with the Soy1066 WGS data filtered for modifying variants using a subset of accessions resistant (FI< 30) to HG 2.5.7 (Race 1) and HG 0. This analysis focused on the candidate CMs. For HG 2.5.7 (Race 1), higher accuracies were achieved for *rhg1-a* (D208E) compared to *rhg1-b* (Q203K) (**Supplementary Table 2**). The missense SNP for L288I, which may be the result of a small indel adjacent to that position, was present in both the *rhg1-a* D208E and *rhg1-b* Q203K alleles and therefore had very high accuracies. Both *Rhg4* (P130R/P200R) and *rhg2* (splice) had very high accuracies for HG 2.5.7 (Race 1), 100%, and 91%, respectively (**Supplementary Table 2**). For the HG 0 (Race 3), *rhg2* (splice) had a very high accuracy 91%, while a lower accuracy was observed for *Rhg4* (78%), which appeared to be the result of the more balanced presence of both *rhg1-b* and *rhg1-a* alleles in resistant accessions (**Supplementary Table 3**). Though a similar accuracy analysis was performed for the GWAS QTNs on chromosomes 2, 14, and 17, the very small number of phenotyped SCN-resistant accessions in the Soy1066 data set along with the multigenic nature of the trait led to insufficient power to identify candidates with high accuracy.

## 4 Discussion

In this study, we provided a detailed overview of the SCN resistance landscape in the University of Missouri soybean breeding programs and the USDA Uniform Soybean Tests—Northern Region (NUST). Overall, through multiple GWASs using various SCN populations with different levels of virulence, we identified seven major QTNs on chromosomes 2, 7, 8, 11, 14, 17, and 18. Further, we successfully linked the QTNs detected in our study to the causal mutations previously reported for *Rhg1*, *rhg2*, and *Rhg4* loci using accuracy calculations. However, our accuracy calculations for QTN02, QTN07, QTN14, and QTN17 were limited by low detection power due to the relatively small number of phenotyped SCN-resistant accessions in the Soy1066 database. Due to the proximity (<300,000 bp) of QTN02, QTN07, and QTN14 to *GmSNAP02*, *GmNSF_RAN07_*, and *GmSNAP14*, respectively, we hypothesize that our identified QTNs potentially represent these candidate genes in this analysis.

Here, we confirmed the PI 88788 (*rhg1-b*) and Peking (*rhg1-a* + *Rhg4*) sources of resistance to HG 0 in the Missouri panel and additionally found that QTN17, in combination with *rhg1-a*, *Rhg4*, and *rhg2*, contributed to resistance against HG 0 (Race 3). The QTN17 reported in this study offers potential for further investigation and introgression into breeding programs for HG 0 (Race 3) resistance. A QTL on chromosome 17 has earlier been reported to be involved in resistance to HG 1.2.5.7 (Wu et al., 2009; Kazi et al., 2010). Further, there is evidence that this genomic region is also involved in resistance to HG 1.3.6.7 in combination with other resistance loci (Schuster et al., 2001), and we show that QTN17 also plays a role in resistance to HG 0 as well as HG 2.5.7 (Races 1 and 5).

Contrastingly, we observed that QTN18b (*rhg1-b*) in combination with QTN07 (*GmNSF_RAN07_*) provided resistance to HG 0 in the NUST panel. Previous studies have demonstrated the coinheritance of *GmNSF_RAN07_* with disease resistance *Rhg1* alleles and its important protective function against *Rhg1 α-SNAP*-related cytotoxicity (Bayless et al., 2016). Here, we expected this locus to be largely fixed in the population and consequently non-significant in the GWAS. However, the locus being significant in this analysis indicates that there are genotypes in the NUST panel that do not harbor SCN resistance. Further, the non-significance of this locus in the Missouri panel is most likely attributed to this locus being fixed in the majority of breeding lines in our study. These contrasting results in our study provide a unique insight into breeding strategies adopted for SCN resistance in Missouri breeding programs versus the rest of the breeding programs in the northern United States.

Epistatic interaction between *rhg1-a* and *rhg2* in combination with *Rhg4* has been demonstrated to confer resistance to virulent HG 2.5.7 (Races 1 and 5) populations (St-Amour et al., 2020; Suzuki et al., 2020; Basnet et al., 2022). We confirmed this in our GWASs for the Missouri panel and additionally found QTN17 and QTN14 to be involved in resistance. These two QTNs helped to impart resistance in combination with *rhg1-a* and *rhg2* to both Races 1 and 5 in the absence of the *Rhg4* resistance allele. All the combinations containing the *rhg1-b* allele were susceptible to these populations in our analysis. Further, we observed only the *Rhg1* locus for resistance to Race 1 in the NUST panel, which hints toward a general lack of effective resistance toward this race in the lines submitted to these trials.

Additionally, an epistatic effect between *rhg1-a* and *rhg2* has previously been shown to impart resistance to HG 1.2.5.7 populations (Guo et al., 2006; Wu et al., 2009; Basnet et al., 2022). In this study, however, we determined that the lines carrying such a combination of associated QTN had a largely bimodal distribution of phenotypes (FI) with a group of susceptible lines and a group of resistant lines. Such distribution of phenotypes could be attributed to multiple alternate alleles of the *Rhg2* locus, where some of these alleles are not involved in resistance to this SCN population. Importantly, this also indicates that other loci are involved in conferring resistance to this virulent SCN population. Partitioning the SCN phenotypes based on markers from our GWAS showed that QTN14 and QTN02, potentially representing *GmSNAP14* and *GmSNAP02*, contributed to resistance against this virulent SCN population in addition to *rhg1-a* and *rhg2*. Further, the *GmSNAP14* and *GmSNAP02* candidate genes, much like *Rhg1* and *Rhg2*, are orthologs of each other, which hints at possible interaction effects between the two loci in the downstream molecular pathways. A previous study has suggested that *GmSNAP14* and *GmSNAP02* play no role in resistance to HG 0 populations (Lakhssassi et al., 2017), yet most recently, *GmSNAP02* has been proven to contribute to SCN resistance in HG 1.2.5.7 populations through a loss-of-function (Usovsky et al., 2023). From our analyses, we conclude that further investigations are needed to delineate the role of the candidate genes *GmSNAP14* and *GmSNAP02* in molecular pathways governing resistance to virulent nematode populations.

The absence of chromosome 8 and 11 QTNs in the NUST panel indicates the low frequency of these important alleles and the overutilization of SCN resistance conferred by the *rhg1-b* allele. The lack of diverse SCN resistance sources in the NUST panel is concerning as resistance governed by the *rhg1-b* allele is not as effective as it once was due to the widespread increase in virulent SCN populations. Contrastingly, diverse modes of resistance to SCN observed in the Missouri panel highlight the historical breeding efforts aimed toward developing SCN-resistant cultivars by the University of Missouri’s southern breeding program. These efforts started in the 1960s as a collaboration between Dr. Edgar Hartwig (USDA, Mississippi) and Dr. Satish Anand (University of Missouri) who aimed to develop breeding lines with Peking-type SCN resistance. Consequently, some of the first SCN-resistant cultivars developed in the United States included ‘Pickett’ (Brim and Ross, 1966), ‘Dyer’ (Epps and Hartwig, 1967), and ‘Custer (Luedders et al., 1968) and carried a combination of *rhg1-a*, *Rhg4*, and *rhg2* resistance loci from the cultivar Peking. Later, PI 437654 and PI 90763 were introduced into the breeding program as sources of SCN resistance, and PI 437654 was backcrossed to Forrest to develop ‘Hartwig’ (PI 543795). Hartwig was the first cultivar reported to be resistant to all known populations of SCN (Anand, 1992). The cultivar Hartwig has been an essential part of the southern breeding program at the Fisher Delta Research, Extension, and Education Center (FDREEC) of the University of Missouri. The next generation of soybean cultivars and germplasm with supreme SCN resistance were released for soybean farmers by Dr. J. Grover Shannon and included S97-1688 (Anand et al., 2004) and S05-11482 (Shannon et al., 2015). These cultivars harbored several sources of SCN resistance genes including Peking, Forrest, PI 437654, and PI 90763. It is important to highlight the unique genetic diversity, high allelic frequency, and unique allelic combinations contributing to SCN resistance in the Missouri panel, which enabled the detection of otherwise rare alleles in this GWAS. The diversity of modes of resistance adopted in the Missouri breeding programs can serve as a valuable resource for other breeding programs in the region to tackle the increase in virulent SCN populations.

Overall in this study, we identified loci governing resistance to virulent SCN populations and described various allelic combinations that could be integrated into breeding programs for SCN resistance. Notably, QTN02 and QTN14 identified in our study are important resources to breed the next generation of SCN-resistant soybeans. Further investigations into the candidate genes, *GmSNAP02* and *GmSNAP14*, associated with these QTNs will enhance our understanding of molecular mechanisms governing SCN resistance. Moreover, the QTN17 reported here holds promise to delineate unique SCN resistance mechanisms along with helping to breed soybean lines with diverse modes of SCN resistance. Finally, we provide a list of breeding lines carrying favorable allelic combinations that could be used in breeding for SCN-resistant germplasm while minimizing the issue of linkage drag associated with trait introgression (**Supplementary Tables 4-7**).

## Data availability statement

The original contributions presented in the study are included in the article/**Supplementary Material**, further inquiries can be directed to the corresponding author.

## Author contributions

AM: Conceptualization, Data curation, Formal analysis, Investigation, Methodology, Visualization, Writing – original draft, Writing – review & editing. KB: Conceptualization, Investigation, Supervision, Writing – original draft, Writing – review & editing. MŠ: Conceptualization, Formal Analysis, Supervision, Writing – original draft, Writing – review & editing. JB: Data curation, Formal Analysis, Investigation, Methodology, Writing – original draft, Writing – review & editing. ED: Data curation, Visualization, Writing – review & editing. CM: Data curation, Writing – review & editing. MU: Data curation, Formal Analysis, Investigation, Supervision, Writing – review & editing. QS: Data curation, Formal Analysis, Writing – review & editing. AL: Data curation, Writing – review & editing. MM: Data curation, Writing – review & editing. GS: Data curation, Funding acquisition, Methodology, Supervision, Writing – review & editing. AS: Conceptualization, Funding acquisition, Project administration, Resources, Supervision, Writing – original draft, Writing – review & editing.
