# Supplementary material for: Cataloging SCN resistance loci in North American public soybean breeding programs
Source: Front Plant Sci. 2023 Nov 20;14:1270546. doi: 10.3389/fpls.2023.1270546 (PMC10694258; doi:10.3389/fpls.2023.1270546)
Supplement: Supplementary file 1 [file DataSheet_1.docx]

Supplementary Material

Cataloging SCN Resistance Loci in North American Public Soybean Breeding Programs

Anser Mahmood^1^, Kristin D. Bilyeu^2^, Mária Škrabišová^3^, Jana Biová^3^, Elizabeth J. De Meyer^1^, Clinton G. Meinhardt^1^, Mariola Usovsky^1^, Qijian Song^4^, Aaron J. Lorenz^5^, Melissa G. Mitchum^6^, Grover Shannon^1^, Andrew M. Scaboo^1*^

*** Correspondence:** Corresponding Author: [scabooa@missouri.edu](mailto:scabooa@missouri.edu)

# Supplementary Data

# Supplementary Figures and Tables

## Supplementary Figures


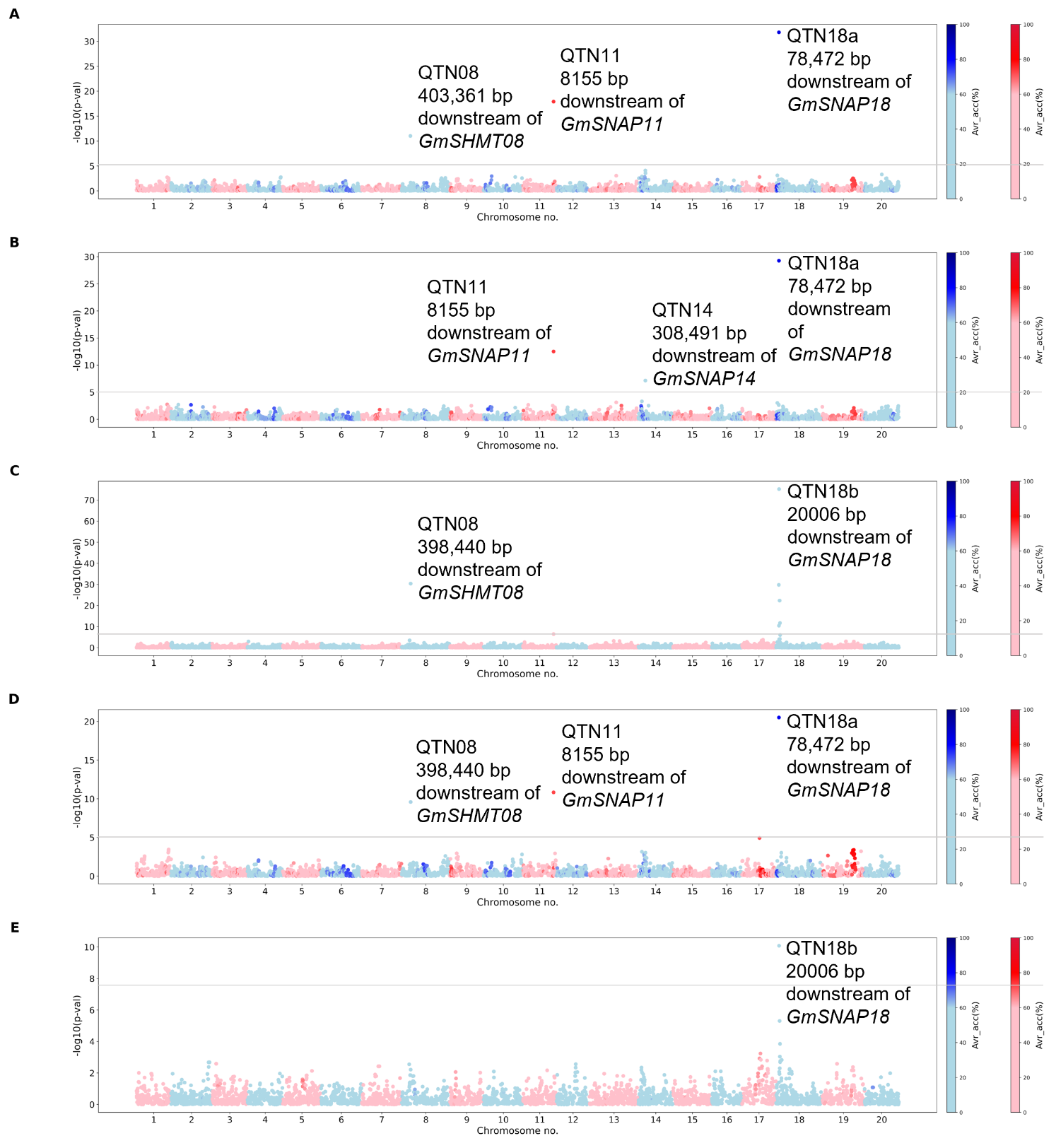


**Supplementary Figure 1.** Manhattan plots generated from genome-wide association study (GWAS) analyses for soybean cyst nematode (SCN) resistance within the Missouri panel using Multi-Locus Mixed-Model (MLMM). The Manhattan plots highlight the average accuracy calculations (using a color scale on the right) for individual SNPs, calculated using the AccuCalc package. **A** Manhattan plot for HG 2.5.7 (Race 1). **B** Manhattan plot for HG 1.2.5.7 (Race 2). **C** Manhattan plot for HG 0 (Race 3). **D** Manhattan plot for HG 2.5.7 (Race 5). **E** Manhattan plot for HG 1.3.6.7 (Race 14).


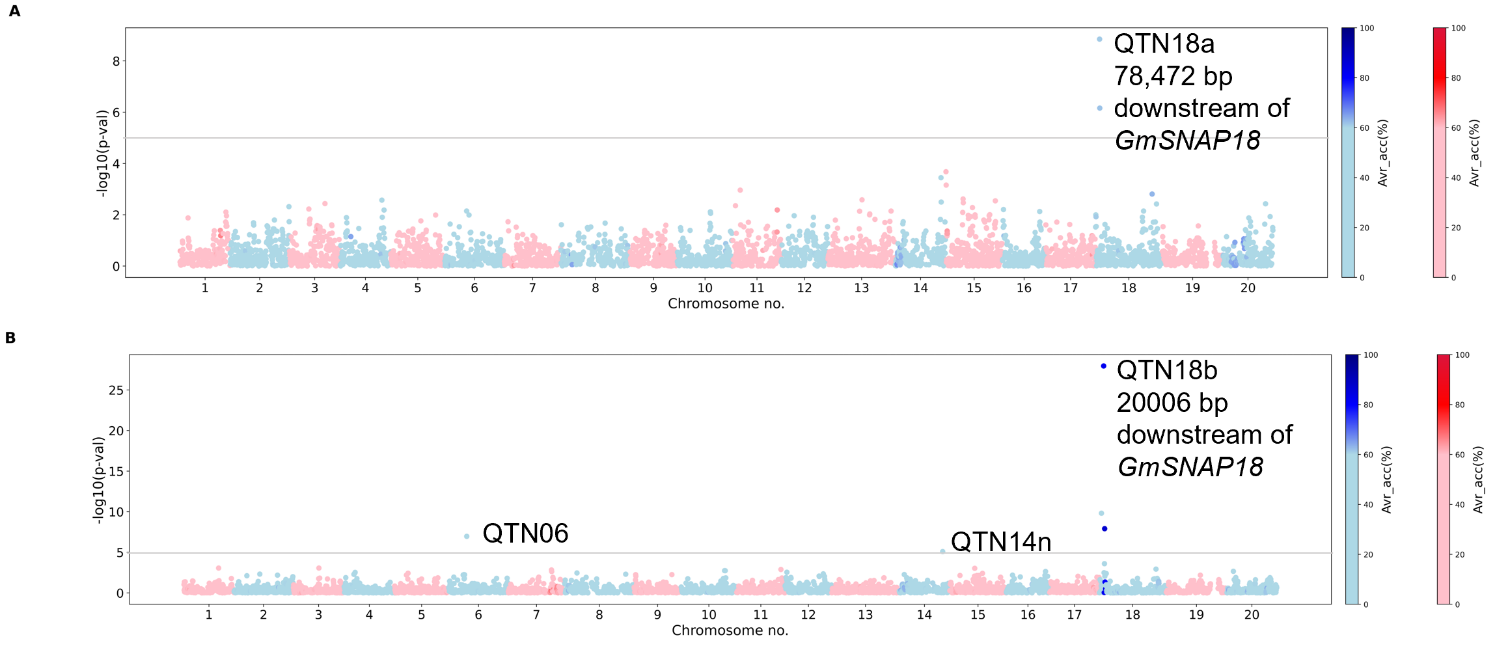


**Supplementary Figure 2.** Manhattan plots generated from GWAS analyses for soybean cyst nematode (SCN) resistance within the NUST panel using Multiple Loci Mixed Model (MLM). The Manhattan plots highlight the average accuracy calculations (using a color scale on the right) for individual SNPs, calculated using the AccuCalc package. **A** Manhattan plot for HG 2.5.7 (Race 1). **B** Manhattan plot for HG 0 (Race 3).





QTN18a

78,472 bp downstream of *GmSNAP18*

QTN18

975,566 bp downstream of *GmSNAP18*

QTN20

**Supplementary Figure 3.** Manhattan plots generated from MLMM-based genome-wide association study (GWAS) analyses for SCN resistance within NUST panel fixed for the alternate allele of the chromosome 18 QTN detected in individual SCN race unfixed GWAS analyses. The lines were fixed for the alternate allele of QTN18a (ss715629144) for HG 2.5.7 (Race 1), while for Race HG 0 (Race 3) the panel was fixed for the alternate allele of QTN18b (ss715629217). The Manhattan plots highlight the average accuracy calculations (using a color scale on the right) for individual SNPs, calculated using the AccuCalc package. **A** Manhattan plot for HG 2.5.7 (Race 1), **B** Manhattan plot for HG 0 (Race 3).

## Supplementary Tables

**Supplementary Table 1. Accuracy of the tagging markers to SCN susceptible (WT, FI>60) and resistant (MUT) binarized phenotypes.** Accuracy analysis of Illumina Infinium BARCSoySNP6K BeadChip DNA markers associated in GWAS. Accuracy is calculated for SCN population HG 2.5.7 (Race 1) and further subsetted for presence of *Rhg* 1 alleles. Chr – chromosome; Avr_Accu – Average accuracy; Avr_Accu order - values sorted from highest to lowest; Acc_WT – WT accuracy; Acc_MUT – MUT accuracy; Count WT – number of accessions with WT phenotype (susceptible to SCN); Count MUT – number of accessions with MUT (SCN-resistant phenotype).

| ***Rhg* locus** | **Chr** | **Position** | **Marker ID** | **Distance to CM (bp)** | **Phenotype** | **Avr_Accu order** | **Avr_Accu (%)** | **Acc_WT (%)** | **Acc_MUT (%)** | **Count WT** | **Count MUT** |
| --- | --- | --- | --- | --- | --- | --- | --- | --- | --- | --- | --- |
| ***Rhg1*** | **18** | 1,562,536 | ss715629144 | -81,107 | FI <30 for Race1 | 1 | 81.4 | 73.33 | 89.47 | 135 | 19 |
|  |  |  |  |  | FI <30 for Race1 with *Rhg1b*^Ref^ | 1 | 83.73 | 73.33 | 94.12 | 135 | 17 |
|  |  |  |  |  | FI <30 for Race1 with *Rhg1a*^Ref^ | 15 | 61.67 | 73.33 | 50.0 | 135 | 4 |
|  |  |  |  |  | FI <30 for Race1 with *Rhg1a/b*^Ref^ | 15 | 61.67 | 73.33 | 50.0 | 135 | 2 |
| ***Rhg4*** | **8** | 7,959,982 | ss715602729 | -401,166 | FI = 0-9 for Race 1 | 2 | 92.59 | 85.19 | 100.0 | 135 | 12 |
|  |  | 8,273,185 | ss715602749 | -87,963 | FI = 0-9 for Race 1 | 12 | 85.19 | 70.37 | 100.0 | 135 | 12 |
| ***rhg2*** | **11** | 32,959,788 | ss715610417 | -10,128 | FI <30 for Race1 | 1 | 89.18 | 88.89 | 89.47 | 135 | 19 |
|  |  |  |  |  | FI <30 for Race3 | 1 | 87.3 | 88.89 | 85.71 | 135 | 28 |

**Supplementary Table 2. Accuracy analysis of *rhg* alleles to SCN Race 1 binarized phenotypes.** Accuracy is calculated for accessions susceptible (WT) and resistant (MUT) to SCN population HG 2.5.7 (Race 1). Chr – chromosome; Avr_Accu – Average accuracy; Avr_Accu order - values sorted from high to lowest; Acc_WT – WT accuracy; Acc_MUT – MUT accuracy; Count WT – number of accessions with WT phenotype (susceptible to SCN); Count MUT – number of accessions with MUT (SCN-resistant phenotype).

| ***Rhg* locus** | ***Rhg* allele** | **Chr** | **Position** | **Effect** | **Phenotype** | **Avr_Accu order** | **Avr_Accu (%)** | **Acc_WT (%)** | **Acc_MUT (%)** | **Count WT** | **Count MUT** |  |
| --- | --- | --- | --- | --- | --- | --- | --- | --- | --- | --- | --- | --- |
| ***Rhg1*** | ***rhg1- a/b* associated mutation** | **18** | 1,645,409 | L288I | FI <30 for Race 1 | 1-3 | 94.74 | 100.0 | 89.47 | 135 | 19 |  |
|  |  |  |  |  | FI <30 for Race 1 with *Rhg1b*^Ref^ | 2-5 | 94.12 | 100.0 | 88.24 | 135 | 17 |  |
|  |  |  |  |  | FI <30 for Race 1 with *Rhg1a*^Ref^ | 3-7 | 75.0 | 100.0 | 50.0 | 135 | 4 |  |
|  | ***rhg1-b*** | **18** | 1,643,643 | Q203K | FI <30 for Race 1 | 27 | 55.26 | 100.0 | 10.53 | 135 | 19 |  |
|  |  |  |  |  |  |  |  |  |  |  |  |  |
|  |  |  |  |  | FI <30 for Race1 with *Rhg1a*^Ref^ | 3-7 | 75.0 | 100.0 | 50.0 | 135 | 4 |  |
|  | ***rhg1-a*** | **18** | 1,643,660 | D208E | FI <30 for Race 1 | 6 | 89.47 | 100.0 | 78.95 | 135 | 19 |  |
|  |  |  |  |  | FI <30 for Race 1 with *Rhg1b*^Ref^ | 2-5 | 94.12 | 100.0 | 88.24 | 135 | 17 |  |
| ***Rhg4*** | ***Rhg4a*** | **8** | 8,361,148 | P200R | FI = 0-9 for Race 1 | 1-2 | 100.0 | 100.0 | 100.0 | 135 | 12 |  |
| ***rhg2*** | ***rhg2*** | **11** | 32,969,916 | Splice donor variant | FI <30 for Race 1 | 1 | 90.66 | 91.85 | 89.47 | 135 | 19 |  |

**Supplementary Table 3. Accuracy analysis of *rhg* alleles to SCN Race 3 binarized phenotypes.** Accuracy is calculated for accessions susceptible (WT) and resistant (MUT) to SCN population HG 0 (Race 3). Chr – chromosome; Avr_Accu – Average accuracy; Avr_Accu order - values sorted from highest to lowest; Acc_WT – WT accuracy; Acc_MUT – MUT accuracy; Count WT – number of accessions with WT phenotype (susceptible to SCN); Count MUT – number of accessions with MUT (SCN-resistant phenotype).

| ***Rhg* locus** | ***Rhg* allele** | **Chr** | **Position** | **Effect** | **Phenotype** | **Avr_Accu order** | **Avr_Accu (%)** | **Acc_WT (%)** | **Acc_MUT (%)** | **Count WT** | **Count MUT** |  |
| --- | --- | --- | --- | --- | --- | --- | --- | --- | --- | --- | --- | --- |
| ***Rhg1*** | ***rhg1-a/b* associated mutation** | **18** | 1,645,409 | L288I | FI <30 for Race 3 | 1-3 | 96.43 | 100.0 | 92.86 | 135 | 28 |  |
|  |  |  |  |  | FI <30 for Race 3 with *Rhg1b*^Ref^ | 1-6 | 91.67 | 100.0 | 83.33 | 135 | 12 |  |
|  |  |  |  |  | FI <30 for Race 3 with *Rhg1a*^Ref^ | 1-4 | 94.44 | 100.0 | 88.89 | 135 | 18 |  |
|  | ***rhg1-b*** | **18** | 1,643,643 | Q203K | FI <30 for Race 3 | 27 | 67.86 | 100.0 | 35.71 | 135 | 28 |  |
|  |  |  |  |  |  |  |  |  |  |  |  |  |
|  |  |  |  |  | FI <30 for Race 3 with *Rhg1a*^Ref^ | 1-6 | 91.67 | 100.0 | 83.33 | 135 | 12 |  |
|  | ***rhg1-a*** | **18** | 1,643,660 | D208E | FI <30 for Race 3 | 9 | 78.57 | 100.0 | 57.14 | 135 | 28 |  |
|  |  |  |  |  | FI <30 for Race 3 with *Rhg1b*^Ref^ | 1-4 | 94.44 | 100.0 | 88.89 | 135 | 18 |  |
| ***Rhg4*** | ***Rhg4a*** | **8** | 8,361,148 | P200R | FI = 0-9 for Race 3 | 33 | 78.26 | 100.0 | 56.52 | 135 | 23 |  |
| ***rhg2*** | ***rhg2*** | **11** | 32,969,916 | Splice donor variant | FI <30 for Race 3 | 1 | 90.57 | 91.85 | 89.29 | 135 | 28 |  |

**Supplementary Table 4.** Missouri panel breeding lines with resistant allelic combinations to HG type 2.5.7 (Race 1)

| Entry | *Rhg1* | *Rhg2* | *Rhg4* | *GmSNAP14* | Chr17 QTN | HG type 2.5.7 (Race1) |
| --- | --- | --- | --- | --- | --- | --- |
| S11-20356 | Rhg-1a | Mut | Ref | Mut | Mut | 11.67 |
| S17-6110 | Rhg-1a | Mut | Ref | Mut | Mut | 4.00 |
| S17-6141 | Rhg-1a | Mut | Ref | Mut | Mut | 4.00 |
| S17-19907 | Rhg-1a | Mut | Ref | Mut | Mut | 10.00 |
| CR17-245 | Rhg-1a | Mut | Ref | Mut | Mut | 16.51 |
| S17-15637 | Rhg-1a | Mut | Ref | Mut | Mut | 12.56 |
| S18-4884 | Rhg-1a | Mut | Ref | Mut | Mut | 20.26 |
| CR17-203 | Rhg-1a | Mut | Ref | Mut | Mut | 12.63 |
| CR17-333 | Rhg-1a | Mut | Ref | Mut | Mut | 16.11 |
| CR17-326 | Rhg-1a | Mut | Ref | Mut | Mut | 13.30 |
| CR17-324 | Rhg-1a | Mut | Ref | Mut | Mut | 11.91 |
| CR17-337 | Rhg-1a | Mut | Ref | Mut | Mut | 10.61 |
| PR17-171 | Rhg-1a | Mut | Ref | Mut | Mut | 10.12 |
| PR17-344 | Rhg-1a | Mut | Ref | Mut | Mut | 13.17 |
| CR17-180 | Rhg-1a | Mut | Ref | Mut | Mut | 2.94 |
| PR17-476 | Rhg-1a | Mut | Ref | Mut | Mut | 1.92 |
| CR17-175 | Rhg-1a | Mut | Ref | Mut | Mut | 0.42 |
| S17-17644 | Rhg-1a | Mut | Ref | Mut | Mut | 1.64 |
| CR17-189 | Rhg-1a | Mut | Ref | Mut | Mut | 10.56 |
| CR17-172 | Rhg-1a | Mut | Ref | Mut | Mut | 4.25 |
| PR17-488 | Rhg-1a | Mut | Ref | Mut | Mut | 0.20 |
| PR17-481 | Rhg-1a | Mut | Ref | Mut | Mut | 0.42 |
| PR17-482 | Rhg-1a | Mut | Ref | Mut | Mut | 0.39 |
| S19-4676 | Rhg-1a | Mut | Ref | Mut | Mut | 43.10 |
| S19-11110 | Rhg-1a | Mut | Ref | Mut | Mut | 18.07 |
| S19-14058 | Rhg-1a | Mut | Ref | Mut | Mut | 1.46 |
| S19-14106 | Rhg-1a | Mut | Ref | Mut | Mut | 18.52 |
| S19-14071 | Rhg-1a | Mut | Ref | Mut | Mut | 12.01 |
| S11-17025 | Rhg-1a | Mut | Ref | Mut | Mut | 4.21 |
| S15-10434 | Rhg-1a | Mut | Ref | Mut | Mut | 1.53 |
| S16-16641 | Rhg-1a | Mut | Ref | Mut | Mut | 0.00 |
| S16-9478 | Rhg-1a | Mut | Ref | Mut | Mut | 2.24 |
| S13-1955 | Rhg-1a | Mut | Ref | Mut | Mut | 32.07 |
| S17-19948 | Rhg-1a | Mut | Mut | Ref | Mut | 7.79 |
| PR18-192 | Rhg-1a | Mut | Mut | Ref | Mut | 5.93 |
| S19-13164 | Rhg-1a | Mut | Mut | Ref | Mut | 42.87 |
| S19-19742 | Rhg-1a | Mut | Mut | Ref | Mut | 4.71 |
| S19-19812 | Rhg-1a | Mut | Mut | Mut | Ref | 2.58 |
| S14-9017 | Rhg-1a | Mut | Mut | Mut | Ref | 1.32 |
| SA19-12541 | Rhg-1a | Mut | Mut | Mut | Ref | 7.84 |
| SA19-15194 | Rhg-1a | Mut | Mut | Mut | Ref | 7.04 |
| SA19-28278 | Rhg-1a | Mut | Mut | Mut | Ref | 3.09 |
| SA19-28336 | Rhg-1a | Mut | Mut | Mut | Ref | 6.87 |
| S17-17797 | Rhg-1a | Mut | Ref | Mut | Ref | 19.00 |
| S18-5852 | Rhg-1a | Mut | Ref | Mut | Ref | 1.02 |
| S18-1042 | Rhg-1a | Mut | Ref | Mut | Ref | 16.27 |
| S18-1025 | Rhg-1a | Mut | Ref | Mut | Ref | 25.19 |
| S17-13496 | Rhg-1a | Mut | Ref | Mut | Ref | 24.00 |
| S17-17824 | Rhg-1a | Mut | Ref | Mut | Ref | 16.05 |
| S18-2157 | Rhg-1a | Mut | Ref | Mut | Ref | 24.76 |
| S18-2195 | Rhg-1a | Mut | Ref | Mut | Ref | 40.31 |
| S18-2175 | Rhg-1a | Mut | Ref | Mut | Ref | 20.05 |
| S18-10555 | Rhg-1a | Mut | Ref | Mut | Ref | 28.54 |
| S18-10560 | Rhg-1a | Mut | Ref | Mut | Ref | 24.55 |
| S18-10569 | Rhg-1a | Mut | Ref | Mut | Ref | 40.31 |
| S18-3869 | Rhg-1a | Mut | Ref | Mut | Ref | 19.74 |
| S18-9847 | Rhg-1a | Mut | Ref | Mut | Ref | 41.64 |
| S18-5956 | Rhg-1a | Mut | Ref | Mut | Ref | 19.74 |
| S18-1639 | Rhg-1a | Mut | Ref | Mut | Ref | 15.96 |
| S19-11186 | Rhg-1a | Mut | Ref | Mut | Ref | 35.47 |
| S19-11254 | Rhg-1a | Mut | Ref | Mut | Ref | 15.60 |
| S19-16063 | Rhg-1a | Mut | Ref | Mut | Ref | 24.69 |
| S19-7867 | Rhg-1a | Mut | Ref | Mut | Ref | 20.20 |
| S19-3084 | Rhg-1a | Mut | Ref | Mut | Ref | 19.19 |
| S19-12613 | Rhg-1a | Mut | Ref | Mut | Ref | 60.00 |
| S16-16814 | Rhg-1a | Mut | Ref | Mut | Ref | 9.42 |
| S13-10590 | Rhg-1a | Mut | Ref | Mut | Ref | 49.40 |
| SA18-9525 | Rhg-1a | Mut | Ref | Mut | Ref | 16.00 |
| SA19-12375 | Rhg-1a | Mut | Ref | Mut | Ref | 60.00 |
| SA19-12549 | Rhg-1a | Mut | Ref | Mut | Ref | 60.00 |
| SA19-12564 | Rhg-1a | Mut | Ref | Mut | Ref | 60.00 |
| SA19-28501 | Rhg-1a | Mut | Ref | Mut | Ref | 60.00 |
| SA19-28587 | Rhg-1a | Mut | Ref | Mut | Ref | 15.00 |
| SA19-7048 | Rhg-1a | Mut | Ref | Mut | Ref | 53.26 |
| SA19-9788 | Rhg-1a | Mut | Ref | Mut | Ref | 15.81 |
| SA19-9915 | Rhg-1a | Mut | Ref | Mut | Ref | 11.11 |
| SA19-9965 | Rhg-1a | Mut | Ref | Mut | Ref | 16.38 |
| S11-16653 | Rhg-1a | Mut | Mut | Ref | Ref | 1.60 |
| S17-17240 | Rhg-1a | Mut | Mut | Ref | Ref | 9.00 |
| S17-17329 | Rhg-1a | Mut | Mut | Ref | Ref | 5.84 |
| PR18-194 | Rhg-1a | Mut | Mut | Ref | Ref | 3.89 |
| PR18-190 | Rhg-1a | Mut | Mut | Ref | Ref | 5.01 |
| S17-1440 | Rhg-1a | Mut | Mut | Ref | Ref | 6.03 |
| PR18-213 | Rhg-1a | Mut | Mut | Ref | Ref | 5.42 |
| PR18-202 | Rhg-1a | Mut | Mut | Ref | Ref | 6.04 |
| PR18-206 | Rhg-1a | Mut | Mut | Ref | Ref | 4.30 |
| S19-19963 | Rhg-1a | Mut | Mut | Ref | Ref | 6.06 |
| S19-19741 | Rhg-1a | Mut | Mut | Ref | Ref | 4.21 |
| S19-19745 | Rhg-1a | Mut | Mut | Ref | Ref | 5.39 |
| S19-19923 | Rhg-1a | Mut | Mut | Ref | Ref | 6.17 |
| S19-19746 | Rhg-1a | Mut | Mut | Ref | Ref | 4.26 |
| SA18-12073 | Rhg-1a | Mut | Mut | Ref | Ref | 9.77 |
| SA18-12149 | Rhg-1a | Mut | Mut | Ref | Ref | 11.34 |

**Supplementary Table 5.** Missouri panel breeding lines with resistant allelic combinations to HG type 1.2.5.7 (Race 2)

| Entry | *Rhg1* | *Rhg2* | *GmSNAP14* | *GmSNAP02* | HG type 1.2.5.7 (Race 2) |
| --- | --- | --- | --- | --- | --- |
| S11-20356 | Rhg-1a | Mut | Mut | Ref | 1.25 |
| S17-1200 | Rhg-1a | Mut | Mut | Ref | 21.00 |
| S18-5852 | Rhg-1a | Mut | Mut | Ref | 0.17 |
| S18-5884 | Rhg-1a | Mut | Mut | Ref | 0.00 |
| S18-2157 | Rhg-1a | Mut | Mut | Ref | 19.05 |
| S18-2195 | Rhg-1a | Mut | Mut | Ref | 11.36 |
| S18-2175 | Rhg-1a | Mut | Mut | Ref | 20.55 |
| CR17-180 | Rhg-1a | Mut | Mut | Ref | 4.01 |
| PR17-476 | Rhg-1a | Mut | Mut | Ref | 10.59 |
| CR17-175 | Rhg-1a | Mut | Mut | Ref | 0.06 |
| S18-3869 | Rhg-1a | Mut | Mut | Ref | 10.53 |
| CR17-172 | Rhg-1a | Mut | Mut | Ref | 6.41 |
| PR17-488 | Rhg-1a | Mut | Mut | Ref | 0.17 |
| PR17-481 | Rhg-1a | Mut | Mut | Ref | 0.42 |
| PR17-482 | Rhg-1a | Mut | Mut | Ref | 2.17 |
| S19-11418 | Rhg-1a | Mut | Mut | Ref | 15.28 |
| S19-16063 | Rhg-1a | Mut | Mut | Ref | 13.09 |
| S19-4657 | Rhg-1a | Mut | Mut | Ref | 0.09 |
| S19-14058 | Rhg-1a | Mut | Mut | Ref | 0.00 |
| S19-12613 | Rhg-1a | Mut | Mut | Ref | 60.00 |
| S11-17025 | Rhg-1a | Mut | Mut | Ref | 0.75 |
| S16-16641 | Rhg-1a | Mut | Mut | Ref | 0.10 |
| S13-10590 | Rhg-1a | Mut | Mut | Ref | 50.03 |

**Supplementary Table 6.** Missouri panel breeding lines with resistant allelic combinations to HG type 0 (Race 3)

| Entry | *Rhg1* | *Rhg4* | *Rhg2* | Chr17 QTN | HG type 0 (Race 3) |
| --- | --- | --- | --- | --- | --- |
| S11-16653 | Rhg-1a | Mut | Mut | Mut | 1.25 |
| S17-1474 | Rhg-1b | Mut | Mut | Mut | 28.00 |
| S17-17329 | Rhg-1a | Mut | Mut | Mut | 0.00 |
| PR18-190 | Rhg-1a | Mut | Mut | Mut | 0.31 |
| S17-19948 | Rhg-1a | Mut | Mut | Mut | 1.29 |
| S17-1440 | Rhg-1a | Mut | Mut | Mut | 0.84 |
| PR18-213 | Rhg-1a | Mut | Mut | Mut | 0.61 |
| PR18-192 | Rhg-1a | Mut | Mut | Mut | 0.53 |
| PR18-202 | Rhg-1a | Mut | Mut | Mut | 0.38 |
| PR18-206 | Rhg-1a | Mut | Mut | Mut | 0.84 |
| PR18-191 | Rhg-1a | Mut | Mut | Mut | 0.46 |
| S19-13164 | Rhg-1a | Mut | Mut | Mut | 30.85 |
| S19-19812 | Rhg-1a | Mut | Mut | Mut | 0.31 |
| S19-19963 | Rhg-1a | Mut | Mut | Mut | 0.78 |
| S19-19741 | Rhg-1a | Mut | Mut | Mut | 1.48 |
| S19-19742 | Rhg-1a | Mut | Mut | Mut | 0.62 |
| S19-19745 | Rhg-1a | Mut | Mut | Mut | 0.54 |
| S19-19746 | Rhg-1a | Mut | Mut | Mut | 1.24 |
| S16-11222 | Rhg-1b | Mut | Mut | Mut | 60.00 |
| S19-18619 | Rhg-1a | Mut | Mut | Ref | 2.10 |
| S14-9017 | Rhg-1a | Mut | Mut | Ref | 3.36 |
| SA18-12073 | Rhg-1a | Mut | Mut | Ref | 4.92 |
| SA18-12086 | Rhg-1a | Mut | Mut | Ref | 6.66 |
| SA18-12149 | Rhg-1a | Mut | Mut | Ref | 5.79 |
| SA18-9556 | Rhg-1b | Mut | Mut | Ref | 8.41 |
| SA19-12541 | Rhg-1a | Mut | Mut | Ref | 3.29 |
| SA19-13585 | Rhg-1b | Mut | Mut | Ref | 11.73 |
| SA19-13736 | Rhg-1b | Mut | Mut | Ref | 14.88 |
| SA19-15142 | Rhg-1a | Mut | Mut | Ref | 3.84 |
| SA19-15194 | Rhg-1a | Mut | Mut | Ref | 3.50 |
| SA19-28278 | Rhg-1a | Mut | Mut | Ref | 3.68 |
| SA19-28289 | Rhg-1b | Mut | Mut | Ref | 14.08 |
| SA19-28336 | Rhg-1a | Mut | Mut | Ref | 5.61 |
| SA19-7232 | Rhg-1b | Mut | Mut | Ref | 12.05 |
| SA19-7424 | Rhg-1b | Mut | Mut | Ref | 12.24 |
| SA19-7472 | Rhg-1b | Mut | Mut | Ref | 14.54 |
| SA20-10273 | Rhg-1b | Mut | Mut | Ref | 11.59 |
| S17-17281 | Rhg-1a | Mut | Ref | Mut | 5.30 |
| S16-8290 | Rhg-1a | Mut | Ref | Mut | 4.57 |
| S19-18527 | Rhg-1a | Mut | Ref | Ref | 7.38 |
| S19-18591 | Rhg-1a | Mut | Ref | Ref | 4.90 |
| S19-3812 | Rhg-1a | Mut | Ref | Ref | 7.77 |
| S19-18584 | Rhg-1a | Mut | Ref | Ref | 15.77 |
| SA18-10539 | Rhg-1a | Mut | Ref | Ref | 9.50 |
| SA18-11523 | Rhg-1a | Mut | Ref | Ref | 8.19 |
| SA18-12148 | Rhg-1b | Mut | Ref | Ref | 14.53 |
| SA19-28270 | Rhg-1b | Mut | Ref | Ref | 16.01 |
| SA19-28420 | Rhg-1b | Mut | Ref | Ref | 13.80 |
| SA19-9012 | Rhg-1b | Mut | Ref | Ref | 9.12 |

**Supplementary Table 7.** Missouri panel breeding lines with resistant allelic combinations to HG type 2.5.7 (Race 5)

| Entry | *Rhg1* | *Rhg2* | *Rhg4* | Chr17 QTN | *GmSNAP14* | HG type 2.5.7 (Race 5) |
| --- | --- | --- | --- | --- | --- | --- |
| S17-19948 | Rhg-1a | Mut | Mut | Mut | Mut | 9.13 |
| PR18-192 | Rhg-1a | Mut | Mut | Mut | Mut | 8.98 |
| S19-19742 | Rhg-1a | Mut | Mut | Mut | Mut | 4.60 |
| S11-16653 | Rhg-1a | Mut | Mut | Ref | Mut | 20.13 |
| S17-17329 | Rhg-1a | Mut | Mut | Ref | Mut | 9.58 |
| PR18-194 | Rhg-1a | Mut | Mut | Ref | Mut | 6.49 |
| PR18-190 | Rhg-1a | Mut | Mut | Ref | Mut | 4.44 |
| PR18-213 | Rhg-1a | Mut | Mut | Ref | Mut | 7.56 |
| PR18-206 | Rhg-1a | Mut | Mut | Ref | Mut | 8.62 |
| S19-18619 | Rhg-1a | Mut | Mut | Ref | Mut | 6.35 |
| S19-19812 | Rhg-1a | Mut | Mut | Ref | Mut | 0.88 |
| S19-19963 | Rhg-1a | Mut | Mut | Ref | Mut | 4.27 |
| S19-19741 | Rhg-1a | Mut | Mut | Ref | Mut | 3.83 |
| S19-19745 | Rhg-1a | Mut | Mut | Ref | Mut | 6.79 |
| S19-19923 | Rhg-1a | Mut | Mut | Ref | Mut | 5.81 |
| S19-19746 | Rhg-1a | Mut | Mut | Ref | Mut | 5.04 |
| S14-9017 | Rhg-1a | Mut | Mut | Ref | Mut | 4.81 |
| S11-20356 | Rhg-1a | Mut | Ref | Mut | Mut | 0.51 |
| S17-6110 | Rhg-1a | Mut | Ref | Mut | Mut | 8.00 |
| S17-6141 | Rhg-1a | Mut | Ref | Mut | Mut | 9.00 |
| CR17-245 | Rhg-1a | Mut | Ref | Mut | Mut | 18.65 |
| S17-15637 | Rhg-1a | Mut | Ref | Mut | Mut | 16.89 |
| S18-5815 | Rhg-1a | Mut | Ref | Mut | Mut | 17.33 |
| CR17-203 | Rhg-1a | Mut | Ref | Mut | Mut | 13.99 |
| CR17-333 | Rhg-1a | Mut | Ref | Mut | Mut | 16.04 |
| CR17-326 | Rhg-1a | Mut | Ref | Mut | Mut | 17.79 |
| CR17-324 | Rhg-1a | Mut | Ref | Mut | Mut | 12.95 |
| CR17-337 | Rhg-1a | Mut | Ref | Mut | Mut | 13.48 |
| PR17-171 | Rhg-1a | Mut | Ref | Mut | Mut | 16.25 |
| PR17-344 | Rhg-1a | Mut | Ref | Mut | Mut | 19.28 |
| CR17-180 | Rhg-1a | Mut | Ref | Mut | Mut | 7.23 |
| PR17-476 | Rhg-1a | Mut | Ref | Mut | Mut | 10.19 |
| CR17-175 | Rhg-1a | Mut | Ref | Mut | Mut | 2.09 |
| S17-17644 | Rhg-1a | Mut | Ref | Mut | Mut | 3.78 |
| CR17-189 | Rhg-1a | Mut | Ref | Mut | Mut | 18.07 |
| CR17-172 | Rhg-1a | Mut | Ref | Mut | Mut | 10.36 |
| PR17-488 | Rhg-1a | Mut | Ref | Mut | Mut | 1.99 |
| PR17-481 | Rhg-1a | Mut | Ref | Mut | Mut | 0.87 |
| PR17-482 | Rhg-1a | Mut | Ref | Mut | Mut | 0.87 |
| S19-4676 | Rhg-1a | Mut | Ref | Mut | Mut | 54.22 |
| S19-11110 | Rhg-1a | Mut | Ref | Mut | Mut | 16.76 |
| S19-14058 | Rhg-1a | Mut | Ref | Mut | Mut | 1.97 |
| S19-14106 | Rhg-1a | Mut | Ref | Mut | Mut | 14.35 |
| S19-14071 | Rhg-1a | Mut | Ref | Mut | Mut | 18.07 |
| S19-14829 | Rhg-1a | Mut | Ref | Mut | Mut | 48.52 |
| S11-17025 | Rhg-1a | Mut | Ref | Mut | Mut | 1.47 |
| S15-10434 | Rhg-1a | Mut | Ref | Mut | Mut | 15.02 |
| S16-16641 | Rhg-1a | Mut | Ref | Mut | Mut | 1.83 |
| S16-5524 | Rhg-1a | Mut | Ref | Mut | Mut | 1.26 |
| S16-9478 | Rhg-1a | Mut | Ref | Mut | Mut | 6.72 |
| S13-1955 | Rhg-1a | Mut | Ref | Mut | Mut | 6.99 |
